# Antioxidant Potential of Adiponectin and Full PPAR-*γ* Agonist in Correcting Streptozotocin-Induced Vascular Abnormality in Spontaneously Hypertensive Rats

**DOI:** 10.1155/2021/6661181

**Published:** 2021-10-14

**Authors:** Sheryar Afzal, Munavvar Abdul Sattar, Edward James Johns, Olorunfemi A. Eseyin, Ali Attiq

**Affiliations:** ^1^Department of Pharmacology & Toxicology, Faculty of Pharmacy, MAHSA University, Selangor, Malaysia; ^2^School of Pharmaceutical Sciences, University Sains Malaysia, Penang, Malaysia

## Abstract

Oxidative stress, which is associated with metabolic and anthropometric perturbations, leads to reactive oxygen species production and decrease in plasma adiponectin concentration. We investigated pharmacodynamically the pathophysiological role and potential implication of exogenously administered adiponectin with full and partial peroxisome proliferator-activated receptor-gamma (PPAR-*γ*) agonists on modulation of oxidative stress, metabolic dysregulation, and antioxidant potential in streptozotocin-induced spontaneously hypertensive rats (SHR). Group I (WKY) serves as the normotensive control, whereas 42 male SHRs were randomized equally into 7 groups (*n* = 6); group II serves as the SHR control, group III serves as the SHR diabetic control, and groups IV, V, and VI are treated with irbesartan (30 mg/kg), pioglitazone (10 mg/kg), and adiponectin (2.5 *μ*g/kg), whereas groups VII and VIII received cotreatments as irbesartan+adiponectin and pioglitazone+adiponectin, respectively. Diabetes was induced using an intraperitoneal injection of streptozotocin (40 mg/kg). Plasma adiponectin, lipid contents, and arterial stiffness with oxidative stress biomarkers were measured using an in vitro and in vivo analysis. Diabetic SHRs exhibited hyperglycemia, hypertriglyceridemia, hypercholesterolemia, and increased arterial stiffness with reduced plasma adiponectin and antioxidant enzymatic levels (*P* < 0.05). Diabetic SHRs pretreated with pioglitazone and adiponectin separately exerted improvements in antioxidant enzyme activities, abrogated arterial stiffness, and offset the increased production of reactive oxygen species and dyslipidemic effects of STZ, whereas the blood pressure values were significantly reduced in the irbesartan-treated groups (all *P* < 0.05). The combined treatment of exogenously administered adiponectin with full PPAR-*γ* agonist augmented the improvement in lipid contents and adiponectin concentration and restored arterial stiffness with antioxidant potential effects, indicating the degree of synergism between adiponectin and full PPAR-*γ* agonists (pioglitazone).

## 1. Introduction

Diabetes mellitus (DM) and hypertension (HTN) are being considered foremost public health and medical issues; therefore, their coexistence has received greater attention because of rising epidemic globally as common chronic diseases, which associate mainly with micro- and macrocardiovascular complications [1], thus accelerating hyperglycemic issues in diabetic individuals [2]. Previous studies also signify the role of hyperglycemia leading to oxidative stress (OS) with endothelial dysfunction in blood vessels of type 1 diabetic patients [3]. Additionally, the concepts of oxidative stress and endothelial dysfunction have gained interest in recent years as contributing factors in the pathogenesis of hypertension and diabetes.

Of note, endothelial dysfunction connects with atherosclerosis progression [4], although hyperglycemia and diabetic complications, as metabolic perturbations, influence the endothelial dysfunction in the first step of vascular changes [5] through complex mechanisms including oxidative stress (OS) and reactive oxygen species (ROS) [6]. Oxidative stress and derivatives of reactive oxygen metabolites significantly aggravate in diabetic states [1, 3], essential hypertension [7], and hyperlipidemia disorders [8], whereas this pathogenesis relates directly to the biological antioxidant capacity of the body [9]. Additionally, recent studies have shown that oxidative stress negatively regulates the adiponectin gene expression [10]; therefore, its concentration in plasma impacts oxidative stress [2, 11].

Adiponectin, an adipokine in the human body [12], serves as a biomarker for the determination of oxidative stress of the body [13]. Adiponectin, upon binding to its receptors, increases oxidation of fatty acids and glucose uptake via activating the peroxisome proliferator-activated receptor-gamma (PPAR-*γ*) ligand pathway [14], thus possessing potential for the treatment of diabetic complications [15] with vascular disorders [16], whereas PPAR-*γ* on activation directly impacts the adiponectin gene transcription [17].

Thiazolidinedione's derivative, pioglitazone, acts as a PPAR-*γ* agonist [18], therapeutically improving insulin resistance and promoting adipocyte differentiation [19]. In addition, the antihypertensive effect of pioglitazone has been ascribed to a reduction in vascular reactivity in terms of vasodilatory action/properties [20] with an increase in plasma adiponectin concentration through PPAR-*γ* activation [21].

In addition, various antihypertensive agents including *β*-blockers, calcium channel blockers, ACE inhibitors, and AT1-antagonists partially mediate their effects by decreasing oxidative stress [22]. There are reports that angiotensin II receptor blockers (ARBs) also possess partial agonistic activity for PPAR-*γ* [23]; therefore, irbesartan increases adiponectin production directly by activating PPAR-*γ*, self-regulating its AT1R blocker characteristics [24]. Moreover, long-term ARB treatment causes a reduction in pulse wave velocity (PWV) [25], thus inhibiting arterial stiffness, independently, of their antihypertensive property [26].

Nonetheless, very less is known about the antioxidant potential of adiponectin in the genetic model of spontaneously hypertensive rats (SHRs) with type 1 diabetic states. In light of the above background, we tried to evaluate pharmacodynamically the pathophysiological role of exogenously administered adiponectin with PPAR-*γ* agonists in attenuating oxidative stress and arterial stiffness with a variation in metabolic and systemic factors including blood pressure, glycemia, and hypertriglyceridemia in streptozotocin- (STZ-) induced SHRs employing both *in vivo* and *in vitro* parameters. The hypothesis also explored whether a potentiating potential or synergistic effect exists between adiponectin with either partial or full PPAR-*γ* agonists, in alleviating oxidative stress caused by STZ in SHRs. Moreover, the relationships between plasma adiponectin and arterial stiffness using pulse wave velocity (PWV) in type 1 diabetic SHRs were also investigated.

## 2. Material and Methods

### 2.1. Animal Grouping and Experimental Protocol

Eight groups of rats were used for this study (*n* = 6). Forty-two spontaneously hypertensive rats (SHR) and six Wistar Kyoto rats (WKY), averaging 230-255 g body weight, divided randomly into eight groups, were kept in stainless, metabolic cages for 3 days for acclimatization purposes and were fed with commercial rat chow (Gold Coin Sdn. Bhd., Penang, Malaysia) with tap water ad libitum in the animal care facility, Universiti Sains Malaysia, Malaysia (where *n* = 6 in each cage), in which six (06) WKYs were used as the control group (WKY+CNT). Forty-two (42) SHRs were divided into 7 groups (where *n* = 6 in each group), whereas thirty-six (36) SHRs (6 groups) received STZ and were treated as per experimental protocol and six (6) SHRs served as the control (SHR+CNT) group. All procedures and animal handling were carried out in accordance with the guidelines research centre “Animal Research and Service Centre (ARASC), USM (main campus),” with ethical approval number: 2012 [28] (352) by the “Animal Ethics Committee, Universiti Sains Malaysia (AECUSM), Malaysia.” The animals used in the experiment were grouped following the treatment protocol:

1. Wistar Kyoto rats: treated with vehicle (WKY+CNT)
2. SHR: treated with vehicle (SHR+CNT)
3. SHR+STZ: SHR treated with streptozotocin serving as the SHR diabetic model
4. SHR+STZ+Irb: given irbesartan (30 mg/kg) by oral gavage for 28 days starting from day 1
5. SHR+STZ+Pio: given pioglitazone (10 mg/kg) orally for 28 days starting from day 1
6. SHR+STZ+Adp: given adiponectin 2.5 *μ*g/kg/day, intraperitoneal, from day 21 to day 28
7. SHR+STZ+Irb+Adp: given irbesartan (30 mg/kg) by oral gavage for 28 days starting from day 1 and adiponectin 2.5 *μ*g/kg/day, intraperitoneal, from day 21 to day 28
8. SHR+STZ+Pio+Adp: given pioglitazone (10 mg/kg) by oral gavage for 28 days starting from day 1 and adiponectin 2.5 *μ*g/kg/day, intraperitoneal, from day 21 to day 28

We prepared a model of type 1 diabetic SHRs using a single intraperitoneal injection (I/P) of (STZ) (Nova Laboratories, Sdn, Bhd, Malaysia), 40 mg/kg body weight, dissolved in citrate buffer (10 mM, pH 4.5) [28], whereas all the STZ-induced SHRs were given glucose (10%) for the first 48 hours after injection to offset the early hypoglycemic shock. Blood glucose levels were evaluated using a standard glucometer (FreeStyle, Abbott, Malaysia), and rats with glucose levels > 300 mg/dL on the 7th day were selected for the experiment. Physiological and metabolic perturbations include body weight, 24 hr water intake, and urine collection and were performed on day 0, to establish the basal variables, followed on days 08, 21, and 28 of the experiment. Systemic hemodynamic parameters including systolic blood pressure (SBP), diastolic blood pressure (DBP), mean arterial pressure (MAP), and heart rate (HR) were measured noninvasively (NIBP) using the CODA equipment (Kent Scientific Corporation, Torrington, CT) on a similar day pattern as for metabolic and physiological indices. Pulse wave velocity (PWV) was measured on the acute study day, *i.e.*, day 28. Urine and blood samples were obtained on similar days' pattern, *i.e.*, days 0, 8, 21, and 28 of the study. The blood samples (2 mL) were collected from the tail vein using a rat restrainer; however, plasma samples were obtained following centrifugation of blood at 3500 rpm for 10 minutes and stored at -30°C for further biochemical analysis for parameters including oxidative and antioxidant biomarkers, plasma levels of cholesterol, triglycerides, and low- and high-density lipoprotein measured values. The estimation of plasma adiponectin concentration was carried out using a quantitative assay max rat adiponectin Elisa kit (Chemtron, Biotechnology Sdn, Malaysia).

### 2.2. Drugs Used in the Experimental Protocol

1. Pioglitazone, (±)-5[4[2(5-ethyl-2-pyridyl)ethoxy]benzyl]-thiazolidine-2,4-dione monohydrochloride (Searle, Pvt, Ltd., Pakistan)
2. Streptozotocin (STZ, Nova Laboratories, Sdn, Bhd., Selangor, Malaysia)
3. Irbesartan (Approvel, Sanofi, Aventis, France)
4. Adiponectin (Chemtron Biotechnology Sdn, Bhd, Malaysia)

A stock solution of pioglitazone (10 mg/mL) and irbesartan (30 mg/mL) was prepared by dissolving their tablets in distilled water, whereas full-length recombinant adiponectin was dissolved in 200 *μ*L phosphate buffer saline [29].

### 2.3. Measurement of *In Vivo* Oxidative Stress and Antioxidant Markers

The collected blood plasma samples before the termination of the acute experiment were subjected to a variety of biochemical analyses in order to access the oxidative and antioxidative status of experimental diabetic SHRs. The levels of plasma oxidative stress biomarkers including malondialdehyde (MDA), antioxidant enzyme activities, *i.e.*, total superoxide dismutase (SOD), nitric oxide (NO), total antioxidative activity (TAC), and glutathione peroxidase (GSH) in collected plasma samples were measured using the spectrophotometric detection kits following the instruction manual provided by Institute of Biological Engineering of Nanjing Jiancheng, Nanjing, China.

### 2.4. Plasma Malondialdehyde

In the biological system, oxygen free radicals can be generated by enzymatic and nonenzymatic reactions. Oxygen free radicals upon generation react with polyunsaturated fatty acids resulting in lipid peroxidation and generate lipid peroxide such as the aldehyde group (malondialdehyde MDA) and ketone and hydroxyl groups with some oxygen free radicals. MDA, a product of lipid peroxidation reactions, is generated as a result of the reaction between free radicals and polyunsaturated fatty acids in the cell membrane [30]. Therefore, evaluation of the MDA concentration in the biological samples could reflect the extent of lipid peroxidation and indirectly signify the extent of cell oxidative state.

### 2.5. Total Superoxide Dismutase

Superoxide dismutase (SOD) plays an important role in cellular environments in the prevention of diseases linked to oxidative stress. Superoxide dismutase (SOD) scavenges the superoxide anion free radicals and protects the cells from being injured from oxidative stress in a biological system. We investigated SOD measurement in blood plasma samples using the method as described by Oyanagui [31].

### 2.6. Nitric Oxide

The universal inter- and intracellular molecule, nitric oxide (NO), is involved in regulating the pathophysiology of CVS. Its biological activity is recognized as EDRF responsible for vasodilatation. It is a gaseous free biological molecule with a half-life of few seconds or less *in vivo*, whereas its altered levels are associated with several pathological conditions like hypertension, hypoxia, and diabetes mellitus. The NO detection kit utilizes the nitrate reductase method and provides an accurate and convenient method for the measurement of total nitrate/nitrite concentration in the biological sample.

### 2.7. Total Antioxidant Capacity

The antioxidant defence consists of enzymatic and nonenzymatic components. The defence system protects the biological system from oxidation through three pathways. Firstly, it eliminates activated oxygen and free radicals, secondly decomposes superoxide to block the oxidation chain, and lastly gets rid of catalytic metal ions [32]. All different antioxidants yield greater protection against attack by nitrogen radicals and reactive oxygen. Hence, total antioxidant capacity (TOC) provides more concise biological information about antioxidant status of an organism compared to that obtained by the measurement of individual components.

### 2.8. Plasma Glutathione

Glutathione is a naturally occurring tripeptide and is a significant component of the antioxidant system and offers protection against oxidative damage and in the detoxification processes in the cell. Glutathione is mostly present in its reduced form (GSH) than in the oxidized form (GSSG). GSH is a cofactor for antioxidant enzymes participating in detoxication mechanisms, e.g., glutathione peroxidase (GSH), glutathione transferase, dehydroascorbate, and reductase. GSH scavenges hydroxyl radicals (HO^·¯^) and singlet oxygen (^1^O_2_) directly, whereas the cell redox state can be determined by using the ratio between GSSG/GSH [33].

### 2.9. Measurement of Plasma Cholesterol, Triglycerides, and Lipoprotein (LDL, HDL) Levels

Triglycerides (ester derivative from fatty acids and glycerol) are transported in plasma by lipoproteins, whereas the excess quantity of carbohydrates converts into triglycerides and deposits in the adipose tissue [34]. We employ the phosphate oxidase/peroxidase method using a biochemical analyzer (ChemWell®, Awareness Technology, Inc., FL, USA) for the measurement of plasma triglyceride in collected plasma samples.

### 2.10. Surgical Intervention for Pulse Wave Velocity (PWV) Measurement

All animals were fasted overnight (12-14 hours) prior to the surgical interventions used for acute surgery. All cannulae and the transducer were filled with heparinized saline (20.0 units/mL). All animals were anaesthetized with an intraperitoneal injection of 60 mg/kg sodium pentobarbitone (Nembutal®, CEVA Sante Animale, Libourne, France). The trachea was cannulated with a PP240 tube to get a clear airway passage to facilitate respiration. The left jugular vein was cannulated with a PP50 cannula, to which a 50 mL syringe on an infusion pump (Perfusor secura FT 50 mL, B. Braun) that delivered normal saline throughout the experiment was attached. The left carotid artery was catheterized with a PP50 tube for the direct measurement of arterial BP via a pressure transducer (P23 ID Gould, Statham Instruments, USA) coupled to a computerized data acquisition system (Powerlab®, AD Instruments, Australia). A midline abdominal incision was carried out to expose the left kidney, and the whole dissection process was done using an electrical cautery knife, and the abdominal contents were moved with great care to the right to get the clear exposure of the left kidney. The left kidney was exposed via a ventral midline incision, and a laser Doppler probe (OxyFlo® Probe, Oxford Ltd., UK) attached to the Powerlab® system was placed on the dorsal surface of the kidney for the direct observation of renal cortical blood perfusion (RCBP) values throughout the experiment. Additionally, the left iliac artery was catheterized with a PP50 cannula and was advanced through the abdominal aorta lying at the entrance of the renal artery, whereas the PP50 cannula was kept patent via infusing saline at 3 mL/hr. A time period of 60 minutes was allowed to stabilize the animals after the completion of the surgical protocol. Blood pressure waves from the two pressure transducers were simultaneously imported and displayed on a data acquisition system at a sampling rate of 400/s for 30 min. The measurement of PWV was done as per our lab technique methodology, described by Swarup et al. [35] and was calculated by dividing the propagation distance (**d**) by propagation time (**t**) and expressed as meters per second.

### 2.11. Propagation Distance and Time

At the completion of the acute surgical procedure, the animal was sacrificed with an overdose of sodium pentobarbitone (200 mg/kg) (Nembutal®, CEVA, France). The full length of the aorta was exposed, and the tip of the two cannulae from the carotid and iliac arteries was identified and marked. A damp silk thread was placed along the contour of the aorta and marked at the tips of the two cannulae, and the distance between these two points was determined. After that, the thread was removed and laid straight for the measurement of the distance between the two marks identified. This pulse wave propagation distance was used to calculate the PWV. The propagation time was determined using a manual “foot to foot” technique. The time consumed by the pulse wave (**t**) to move from the aortic arch to the abdominal aorta was measured manually by the time delay between the upstrokes (foot) of each pressure wave front. The average of 10 normal consecutive cardiac cycles was used to calculate the propagation time. Any abnormal waveform within the cycles measured was rejected, and the next viable waveform was measured. The manual foot to foot technique is considered a reliable method for determining PWV [25, 35]. At the termination of the study, all animals used in the experiment were disposed of in accordance with the guidelines of the Animal Ethics Committee of Universiti Sains Malaysia, Malaysia.

### 2.12. Statistical Analysis

The statistical analysis was performed using GraphPad Prism® version 5.00 for Windows (GraphPad Software, San Diego, California, USA). Metabolic parameters including body weight, blood glucose level, and plasma adiponectin concentration and the hemodynamics parameters during the treatment period were analyzed using repeated measures one-way ANOVA followed by the Bonferroni *post hoc* test. Data expressed as the mean ± SEM and differences between the means were considered significant at the 5% level.

## 3. Results

### 3.1. Biochemical and Metabolic Indices

The mean values for metabolic indices including body weight, fluid intake, urine output, and blood glucose concentration of all eight experimental groups were measured on four occasions during the study period, i.e., on day 0, day 8, day 21, and day 28 (Table 1). The initial body weight did not significantly change among all groups including WKY and SHR controls on all four days of observation (**P** > 0.05.) However, the respective body weights of the control groups (WKY+CNT, SHR+CNT) significantly increased on days 8, 21, and 28 as compared to day 0 (**P** < 0.05). As the study progressed, the body weight of the SHR diabetic control (SHR+STZ) and SHR diabetic treated groups including SHR+STZ+Irb, SHR+STZ+Pio, SHR+STZ+Adp, SHR+STZ+Adp+Irb, and SHR+STZ+Adp+Pio follows a significantly decreasing body weight pattern with the duration of diabetes, irrespective of various treatments as compared to day 0 and control groups on days 8, 21, and 28 of the study (**P** < 0.05) (Table 1).

There was no significant difference in fluid intake in the WKY+CNT group (**P** > 0.05), but it remained significantly lower in the SHR+CNT group as compared to the WKY control group on all 4 days of observation (**P** < 0.05). However, in the STZ-induced diabetic model, the SHR+STZ group showed higher water intake on days 8, 21, and 28 as compared to day 0. Similarly, SHR diabetic treated groups exhibited polydipsia as compared to the SHR+CNT group on days 8, 21, and 28 (**P** < 0.05). No significant difference was observed in separate and combined treatment of adiponectin with either irbesartan or pioglitazone (SHR+STZ+Adp, SHR+STZ+Adp+Irb, and SHR+STZ+Adp+Pio) on respective days as compared to SHR+STZ (**P** > 0.05) (Table 1).

Similarly, mean values of the urine flow rate of all experimental groups were observed which was significantly lower in the SHR+CNT as compared to the WKY+CNT group on all 4 days of observation. Contrary to the SHR+CNT group, SHR+STZ-treated rats showed polyuria on days 8, 21, and 28 (**P** < 0.05). However, the SHR+STZ+Irb and SHR+STZ+Pio groups did not show a significant difference on days 8, 21, and 28 (**P** > 0.05), whereas increased significantly in the SHR+STZ+Adp, SHR+STZ+Irb+Adp, and SHR+STZ+Pio+Adp groups on day 28 only as compared to the SHR+STZ group and statistically with greater values in SHR+STZ+Pio+Adp as compared to the SHR+STZ+ADP and SHR+STZ+Irb+Adp groups (**P** < 0.05) (Table 1).

All STZ-administered animals developed diabetes resulting in a significant rise in blood glucose levels of the SHR+STZ versus SHR+CNT group (*P* < 0.05), whereas no significant difference was observed between the WKY and SHR control groups on all four days (*P* > 0.05). Similarly, the SHR+STZ- and SHR+STZ-treated groups showed a significant increase in blood glucose values on days 8, 21, and 28 as compared to the SHR+CNT group (*P* < 0.05). However, statistically, there was no significant effect on the blood glucose levels with any set of treatments during the experiment (*P* > 0.05) (Table 1).

### 3.2. Systemic Hemodynamic

As per the study protocol, baseline values and the changes in the systolic blood pressure (SBP), diastolic blood pressure (DBP), mean arterial pressure (MAP), and heart rate (HR) of eight groups of experimental rats were measured by the tail cuff method on days 0, 8, 21, and 28 of the study (Table 2). We observed that SBP and MAP were significantly higher in SHR+CNT as compared to WKY+CNT on all 4 days of observation (**P** < 0.05), whereas the SHR+STZ groups exhibited increased SBP and MAP values as compared to SHR+CNT on days 21 and 28 only (**P** > 0.05). The SHR+STZ+Irb and SHR+STZ+Pio groups showed a significant decrease in SBP and MAP values on days 21 and 28 and SHR+STZ+Adp on day 28 only as compared to the SHR+STZ group (**P** < 0.05). Interestingly, the SHR+STZ+Irb+Adp group expressed greater significant reduction in SBP and MAP on day 28 as compared to other treatments used in the study (**P** < 0.05), and the values obtained were comparable to the WKY+CNT group (Table 2).

In addition, after induction of diabetes, the mean values of DBP were significantly higher in SHR+CNT as compared to WKY+CNT (*P* < 0.05), but no significant difference was observed in the SHR+CNT and SHR+STZ groups (*P* > 0.05) on similar days of observation, whereas a significant decrease in DBP was observed in the SHR+STZ+Irb and SHR+STZ+Pio groups on day 21 and SHR+STZ+Adp on day 28 only as compared to the SHR+STZ+CNT group (*P* < 0.05). Furthermore, DBP of the SHR+STZ+Irb, SHR+STZ+Pio, SHR+STZ+Adp, SHR+STZ+Irb+Adp, and SHR+STZ+Pio+Adp groups significantly further decrease on day 28 (*P* < 0.05), with a greater extent of reduction in the SHR+STZ+Irb+Adp group in comparison to the SHR+STZ+Irb, SHR+STZ+Adp, and SHR+STZ+Pio+Adp groups (*P* < 0.05) (Table 2).

The heart rate of all groups was observed on the same pattern of days, *i.e.*, days 0, 8, 21, and 28. The heart rate of the SHR+CNT group remained significantly higher as compared to WKY+CNT on all four points of observation. Moreover, the heart rate of the SHR+STZ+CNT group was significantly higher as compared to SHR+CNT on days 21 and 28 (**P** < 0.05). However, treating diabetic SHRs significantly reduced the heart rate in SHR+STZ+Irb, SHR+STZ+Pio, and SHR+STZ+Adp as compared to SHR+CNT on day 28 (**P** < 0.05), whereas the values obtained in SHR+STZ+Adp were of greater extent as compared to the SHR+STZ+Irb and SHR+STZ+Pio groups. No significant effect was observed in the case of combined treatment of adiponectin with either irbesartan or pioglitazone (**P** > 0.05) (Table 2).

### 3.3. Plasma Adiponectin and Lipid Profile Determination

**Figure 1:**
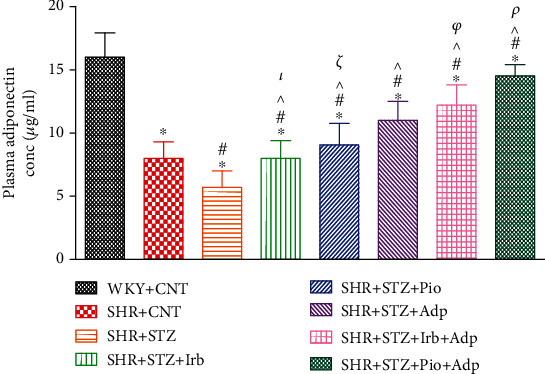
Plasma adiponectin concentration in WKY, SHR diabetic control, and SHR diabetic treated rats. The values are presented as the mean ± SEM (*n* = 6) in each group and were analyzed by one-way ANOVA followed by the *Bonferroni* post hoc test. Values with *P* < 0.05 were considered statistically significant. ^∗^*P* < 0.05 vs. WKY; ^#^*P* < 0.05 vs. SHR. ^^^*P* < 0.05 vs. SHR+STZ+Irb, SHR+STZ+Pio, SHR+STZ+Adp, SHR+STZ+Irb+Adp, and SHR+STZ+Pio+Adp groups in comparison to SHR+STZ. *τ* indicates significant difference between SHR+STZ+Irb and SHR+STZ+Adp. *ξ* indicates significant difference between SHR+STZ+Pio and SHR+STZ+Adp. *ρ* indicates significant difference between SHR+STZ+Adp and SHR+STZ+Pio+Adp. *φ* indicates significant difference between SHR+STZ+Adp and SHR+STZ+Pio+Adp.

Plasma adiponectin concentration and lipid profile were measured on day 28 only in the SHR and SHR diabetic pretreated groups. A significant decrease in plasma adiponectin was observed in SHR+STZ as compared to the WKY+CNT and SHR+CNT groups (**P** < 0.05). The diabetic SHRs treated with irbesartan (30 mg/kg/day), pioglitazone (10 mg/kg/day), and adiponectin (2.5 *μ*g/kg/day) expressed a significant increase in plasma adiponectin concentration as compared to the SHR+STZ+CNT group (**P** < 0.05). Moreover, the combined treatment of adiponectin in the SHR+STZ+Irb+Adp and SHR+STZ+Pio+Adp groups significantly increased plasma concentration of adiponectin as compared to their separate treatments (**P** < 0.05); however, a greater extent of increase in SHR+STZ+Pio+Adp was observed as compared to the SHR+STZ+Irb+Adp group (**P** < 0.05) (Figure 1).

As far as the lipid profile of SHR diabetic treated groups is concerned, SHR+STZ showed a significant increase in triglycerides, low-density lipoproteins, and total serum cholesterol and a decrease in high-density lipoproteins as compared to the SHR+CNT group (Table 3) (**P** < 0.05). Interestingly, adiponectin treatment (SHR+STZ+Adp) caused a significant improvement in all these parameters (**P** < 0.05), whereas the combination of adiponectin with pioglitazone (SHR+STZ+Pio+Adp) caused a greater and significant decrease in triglycerides, low-density lipoproteins, and total serum cholesterol with increases in high-density lipoproteins as compared to either their separate treatment or combination of adiponectin with irbesartan (**P** < 0.05), thus improving the lipid contents of diabetic treated SHRs (Table 3).

### 3.4. Pulse Wave Velocity and Renal Cortical Blood Perfusion

**Figure 2:**
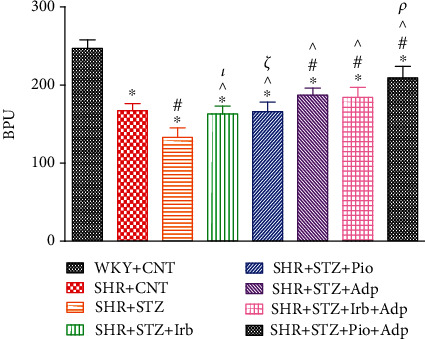
Baseline renal cortical blood perfusion of WKY, SHR diabetic control, and SHR diabetic treated rats. The values are presented as the mean ± SEM (*n* = 6) in each group and were analyzed by one-way ANOVA followed by *Bonferroni* post hoc test. Values with *P* < 0.05 were considered statistically significant. ^∗^*P* < 0.05 versus WKY; ^#^*P* < 0.05 versus SHR; ^^^*P* < 0.05 vs. SHR+STZ+Irb, SHR+STZ+Pio, SHR+STZ+Adp, SHR+STZ+Irb+Adp, and SHR+STZ+Pio+Adp groups in comparison to SHR+STZ. *τ* indicates significant difference between SHR+STZ+Irb and SHR+STZ+Adp. *ξ* indicates significant difference between SHR+STZ+Pio and SHR+STZ+Adp. *ρ* indicates significant difference between SHR+STZ+Adp and SHR+STZ+Pio+Adp.

Recordings for the pulse wave velocity (PWV) and renal cortical blood perfusion (RCBP) for groups including SHR control, STZ-induced diabetic SHRS, and SHR diabetic treated groups were determined during the acute surgical intervention. The RCBP in SHR+STZ was lower as compared to the WKY+CNT and SHR+CNT groups (133 ± 12 vs. 247 ± 11 and 167 ± 9 bpu), respectively (**P** < 0.05). The SHR diabetic treated groups (SHR+STZ+Irb, SHR+STZ+Pio, and SHR+STZ+Adp) showed significantly higher RCBP as compared to the SHR+STZ group (163 ± 9, 166 ± 12, and 187 ± 9 vs. 133 ± 12 bpu), respectively (**P** < 0.05). Moreover, RCBP in the SHR+STZ+Adp group was significantly higher as compared to irbesartan and pioglitazone separate treatments but still remained significantly lower as compared to the WKY+CNT group. The combined treatment in SHR+STZ+Pio+Adp further increased RCBP (209 ± 12 bpu) and was statistically higher as compared to the SHR+STZ+Irb+Adp group (194 ± 6 bpu) (**P** < 0.05) (Figure 2).

**Figure 3:**
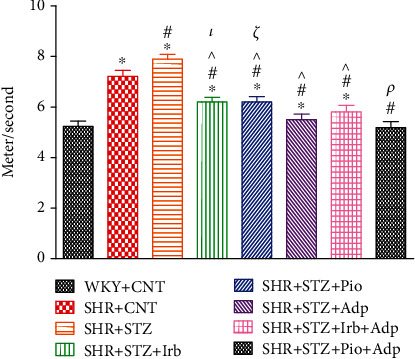
Pulse wave velocity of WKY, SHR diabetic control, and SHR diabetic treated rats. The values are presented as the mean ± SEM (*n* = 6) in each group and were analyzed by one-way ANOVA followed by *Bonferroni* post hoc test. Values with *P* < 0.05 were considered statistically significant. ^∗^*P* < 0.05 versus WKY; ^#^*P* < 0.05 versus SHR; ^^^*P* < 0.05 vs. SHR+STZ+Irb, SHR+STZ+Pio, SHR+STZ+Adp, SHR+STZ+Irb+Adp, and SHR+STZ+Pio+Adp groups in comparison to SHR+STZ. *τ* indicates significant difference between SHR+STZ+Irb and SHR+STZ+Adp. *ξ* indicates significant difference between SHR+STZ+Pio and SHR+STZ+Adp. *ρ* indicates significant difference between SHR+STZ+Adp and SHR+STZ+Pio+Adp.

Moreover, it was observed that the pulse wave velocity (PWV) of SHR+CNT was significantly higher as compared to the WKY+CNT group, whereas PWV of SHR+STZ was significantly higher compared to the SHR+CNT group. This increase in PWV was blunted in SHR+STZ+Irb (6.17 ± 0.17 m/s), SHR+STZ+Pio+Adp (6.14 ± 0.21 m/s), and SHR+STZ+Adp (5.49 ± 0.22 m/s). Furthermore, the tendency to decrease PWV in the adiponectin-treated group was more as compared to the separate irbesartan and pioglitazone groups. Adiponectin with pioglitazone in SHR+STZ+Pio+Adp further reduced PWV and reached the level of the WKY+CNT group (5.27 ± 0.31 m/s) (**P** < 0.05) (Figure 3).

### 3.5. Antioxidant Biomarkers

#### 3.5.1. Plasma Total Superoxide Dismutase and Malondialdehyde

**Figure 4:**
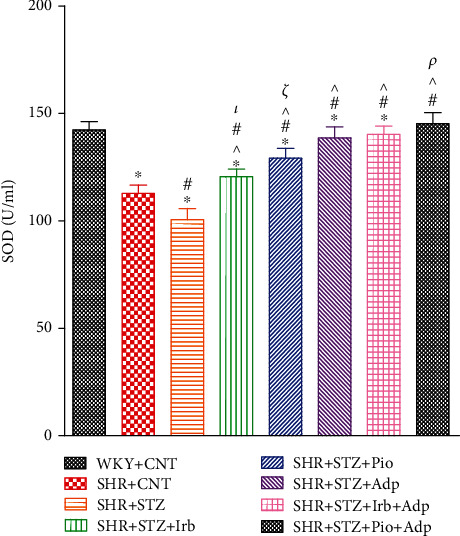
Plasma total superoxide dismutase levels of WKY, SHR diabetic control, and SHR diabetic treated rats. The values are presented as the mean ± SEM (*n* = 6) in each group and were analyzed by one-way ANOVA followed by *Bonferroni post hoc* test. Values with *P* < 0.05 were considered statistically significant. ^∗^*P* < 0.05 versus WKY; ^#^*P* < 0.05 versus SHR; ^^^*P* < 0.05 vs. SHR+STZ+Irb, SHR+STZ+Pio, SHR+STZ+Adp, SHR+STZ+Irb+Adp, and SHR+STZ+Pio+Adp groups in comparison to SHR+STZ. *τ* indicates significant difference between SHR+STZ+Irb and SHR+STZ+Adp. *ξ* indicates significant difference between SHR+STZ+Pio and SHR+STZ+Adp. *ρ* indicates significant difference between SHR+STZ+Adp and SHR+STZ+Pio+Adp.

The plasma total superoxide dismutase (T-SOD) of all experimental groups including diabetic control SHRs and diabetic treated SHRs was measured. The plasma T-SOD of SHR+CNT was significantly lower as compared to WKY+CNT (108.75 ± 3.9 vs. 145.50 ± 3.87 U/mL) (**P** < 0.05), whereas STZ+STZ expressed significantly lower values as compared to the SHR+CNT group (100.58 ± 4.77 vs. 108.75 ± 3.9 U/mL) (**P** < 0.05), which significantly increased in the SHR+STZ+Irb, SHR+STZ+Pio, and SHR+STZ+Adp groups as compared to the SHR+STZ group (119.14 ± 2.68, 125.52 ± 4.51, and 138.56 ± 3.97 vs. 100.58 ± 4.77 U/mL), respectively (**P** < 0.05). The SHR+STZ+Adp showed significantly higher T-SOD values as compared to the SHR+STZ+Irb and SHR+STZ+Pio groups. The combined treatment of adiponectin with pioglitazone in SHR+STZ+Pio+Adp further increases T-SOD values (146.27 ± 5.01 U/mL) and reaches the level of WKY+CNT (**P** > 0.05), as compared to the SHR+STZ+Irb+Adp group which did not show a significant difference to the SHR+STZ+Adp group (143.25 ± 3.81 U/mL) (**P** > 0.05) (Figure 4).

**Figure 5:**
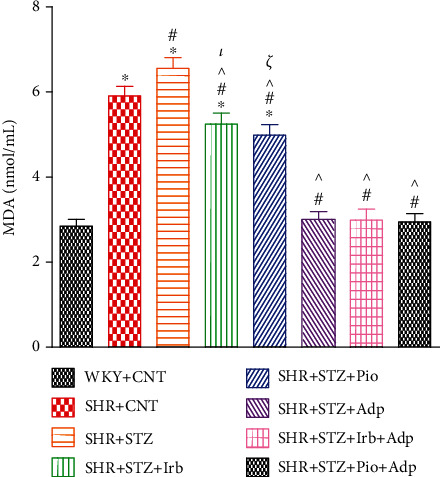
Plasma malondialdehyde levels of WKY, SHR diabetic control, and SHR diabetic treated rats. The values are presented as the mean ± SEM (*n* = 6) in each group and were analyzed by one-way ANOVA followed by *Bonferroni post hoc* test. Values with *P* < 0.05 were considered statistically significant. ^∗^*P* < 0.05 versus WKY; ^#^*P* < 0.05 versus SHR; ^^^*P* < 0.05 vs. SHR+STZ+Irb, SHR+STZ+Pio, SHR+STZ+Adp, SHR+STZ+Irb+Adp, and SHR+STZ+Pio+Adp groups in comparison to SHR+STZ. *τ* indicates significant difference between SHR+STZ+Irb and SHR+STZ+Adp. *ξ* indicates significant difference between SHR+STZ+Pio and SHR+STZ+Adp.

We also obtained the plasma malondialdehyde (MDA) levels of these experimental groups, which were significantly higher in SHR+CNT as compared to WKY+CNT (5.91 ± 0.22 vs. 2.85 ± 0.19 nmol/mL) (**P** < 0.05), whereas plasma MDA levels in SHR+STZ were significantly higher as compared to the SHR+CNT group (6.61 ± 0.25 vs. 5.91 ± 0.23 nmol/mL). The separate treatments with either irbesartan or pioglitazone (SHR+STZ+Irb, SHR+STZ+Pio) significantly reduced MDA concentrations as compared to the SHR+CNT group (5.25 ± 0.25 and 4.99 ± 0.21 vs. 6.61 ± 0.25 nmol/ml), respectively (**P** < 0.05). Furthermore, treatment with adiponectin (SHR+STZ+Adp) and its combination with the irbesartan (SHR+STZ+Irb+Adp) or pioglitazone (SHR+STZ+Pio+Adp) groups further significantly decreased the plasma MDA concentration (3.01 ± 0.17, 2.99 ± 0.11, and 2.95 ± 0.01 nmol/L), respectively (**P** < 0.05). There was no significant difference between the SHR+STZ+Adp group as compared to the SHR+STZ+Irb+Adp and SHR+STZ+Pio+Adp groups (**P** > 0.05) (Figure 5).

#### 3.5.2. The Plasma Nitric Oxide and Total Antioxidant Capacity

**Figure 6:**
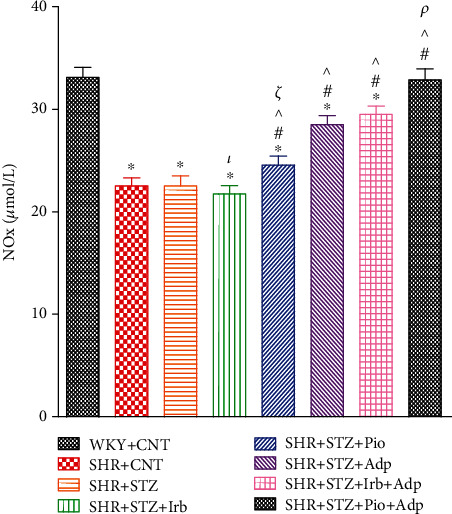
Plasma nitric oxide levels of WKY, SHR diabetic control, and SHR diabetic treated rats. The values are presented as the mean ± SEM (*n* = 6) in each group and were analyzed by one-way ANOVA followed by *Bonferroni post hoc* test. Values with *P* < 0.05 were considered statistically significant. ^∗^*P* < 0.05 versus WKY; ^#^*P* < 0.05 versus SHR; ^^^*P* < 0.05 vs. SHR+STZ+Irb, SHR+STZ+Pio, SHR+STZ+Adp, SHR+STZ+Irb+Adp, and SHR+STZ+Pio+Adp groups in comparison to SHR+STZ. *τ* indicates significant difference between SHR+STZ+Irb and SHR+STZ+Adp. *ξ* indicates significant difference between SHR+STZ+Pio and SHR+STZ+Adp. *ρ* indicates significant difference between SHR+STZ+Adp and SHR+STZ+Pio+Adp.

The plasma nitric oxide (NOx) levels were estimated by measuring the total nitrate/nitrite concentrations in plasma. We observed that the plasma NO level of SHR+CNT was significantly lower as compared to the WKY+CNT group (22.54 ± 0.77 vs. 33.12 ± 0.97 *μ*mol/L), whereas the plasma NO level of SHR+STZ was significantly lower as compared to the SHR+CNT group (20.51 ± 0.86 vs. 22.54 ± 0.77 *μ*mol/L), respectively (**P** < 0.05). Interestingly, pioglitazone and adiponectin single treatments significantly increased plasma NO levels in the SHR+STZ+Pio and SHR+STZ+Adp groups as compared to the SHR+STZ and SHR+STZ+Irb groups (23.56 ± 0.65 and 28.52 ± 0.39 vs. 20.51 ± 0.86 and 21.70 ± 0.71 *μ*mol/L), respectively (**P** < 0.05). The combined treatment of adiponectin with pioglitazone (SHR+STZ+Pio+Adp) further increased the plasma NO level (32.77 ± 0.88 *μ*mol/L) (**P** < 0.05) and was comparable to the WKY+CNT group (Figure 6).

**Figure 7:**
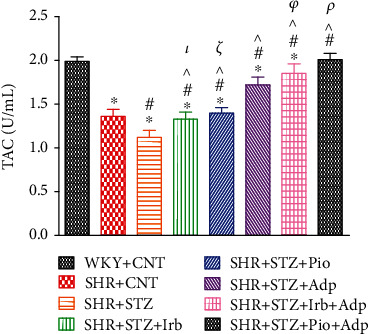
Plasma total antioxidant capacity of WKY, SHR diabetic control, and SHR diabetic treated rats. The values are presented as the mean ± SEM (*n* = 6) in each group and were analyzed by one-way ANOVA followed by *Bonferroni* post hoc test. Values with *P* < 0.05 were considered statistically significant. ^∗^*P* < 0.05 versus WKY; ^#^*P* < 0.05 versus SHR; ^^^*P* < 0.05 vs. SHR+STZ+Irb, SHR+STZ+Pio, SHR+STZ+Adp, SHR+STZ+Irb+Adp, and SHR+STZ+Pio+Adp groups in comparison to SHR+STZ. *τ* indicates significant difference between SHR+STZ+Irb and SHR+STZ+Adp. *ξ* indicates significant difference between SHR+STZ+Pio and SHR+STZ+Adp. *ρ* indicates significant difference between SHR+STZ+Adp and SHR+STZ+Pio+Adp. *φ* indicates significant difference between SHR+STZ+Adp to SHR+STZ+Pio+Adp.

Our observations also recorded significantly decreased values for total antioxidant capacity (TAC) in SHR+CNT as compared to the WKY+CNT group (1.37 ± 0.09 vs. 1.99 ± 0.05 U/mL) (**P** < 0.05), whereas the plasma TAC values in SHR+STZ further significantly reduced as compared to the SHR+CNT group (1.12 ± 0.07 vs. 1.37 ± 0.09 U/mL) (**P** < 0.05). Moreover, treated SHRs with either irbesartan (SHR+STZ+Irb) or pioglitazone (SHR+STZ+Pio) caused significantly increased TAC values as compared to the SHR+STZ group (1.33 ± 0.08 and 1.39 ± 0.05 vs. 1.12 ± 0.07 **U**/**mL**), respectively (**P** < 0.05). The plasma TAC values significantly increased in the SHR+STZ+Adp, SHR+STZ+Irb+Adp, and SHR+STZ+Pio+Adp groups (1.70 ± 0.09, 1.85 ± 0.11, and 2.01 ± 0.07 U/mL), respectively (**P** < 0.05), with greater values obtained in SHR+STZ+Pio+Adp (2.01 ± 0.07 U/mL), and were comparable to the WKY+CNT group (1.99 ± 0.05 U/mL) (**P** < 0.05) (Figure 7).

#### 3.5.3. Plasma Glutathione

**Figure 8:**
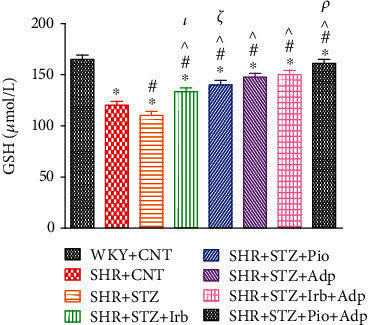
Plasma glutathione level of WKY, SHR diabetic control, and SHR diabetic treated rats. The values are presented as the mean ± SEM (*n* = 6) in each group and were analyzed by one-way ANOVA followed by *Bonferroni post hoc* test. Values with *P* < 0.05 were considered statistically significant. ^∗^*P* < 0.05 versus WKY; ^#^*P* < 0.05 versus SHR; ^^^*P* <0.05 vs. SHR+STZ+Irb, SHR+STZ+Pio, SHR+STZ+Adp, SHR+STZ+Irb+Adp, and SHR+STZ+Pio+Adp groups in comparison to SHR+STZ. *τ* indicates significant difference between SHR+STZ+Irb and SHR+STZ+Adp. *ξ* indicates significant difference between SHR+STZ+Pio and SHR+STZ+Adp. *ρ* indicates significant difference between SHR+STZ+Adp and SHR+STZ+Pio+Adp.

In the last, we also measured plasma glutathione (GSH) in SHR diabetic treated groups, which showed significantly lower values in SHR+CNT as compared to the WKY+CNT group (120.19 ± 3.85 vs. 160.08 ± 4.10 *μ*mol/L) (*P* < 0.05) (Figure 8). However, SHR+STZ significantly reduced GSH values as compared to the SHR+CNT group (110.23 ± 3.77*vs.*120.19 ± 3.85 *μ*mol/L) (*P* < 0.05). Single treatments with irbesartan, pioglitazone, and adiponectin significantly increased GSH values in SHR+STZ+Irb, SHR+STZ+Pio, and SHR+STZ+Adp as compared to the SHR+STZ group (133.49 ± 3.77, 139.22 ± 3.66, and 145.49 ± 5.13 vs.110.21 ± 3.77 *μ*mol/L), respectively (*P* < 0.05). The combined treatment of adiponectin with pioglitazone in SHR+STZ+Pio+Adp further increased GSH values (156.27 ± 3.77) as compared to the SHR+STZ+Irb+Adp group (150.25 ± 4.77 *μ*mol/L) (*P* < 0.05) but was not comparable to the WKY control group (160.08 ± 4.10 *μ*mol/L) (Figure 8).

## 4. Discussion

To the best of our knowledge, this study is among few investigating the pathophysiological role and impact of exogenously administered adiponectin with PPAR-*γ* agonists in streptozotocin- (STZ-) induced spontaneously hypertensive rats (SHRs) by measuring *in vivo* and *in vitro* antioxidant potential, plasma lipid contents, and glycemic and endogenous adiponectin levels with systemic and renal blood pressure measurements. Our results indicate that the continuous administration of STZ had led to vascular abnormalities and impaired endothelial functions with decreased plasma adiponectin concentration. The present study also assessed the relationship between pulse wave velocity (PWV) and oxidative stress markers. Our study indicates that STZ administration leads to a complex mechanism of diabetes and hypertension development, possibly due to the enhanced oxidative stress, indicated by increased MDA and decreased plasma SOD, NOx, and TOC levels. The 28 days of the study period, pharmacodynamically, revealed that adiponectin, as a biomarker, in combination with full PPAR-*γ* agonist, pioglitazone, abrogates oxidative stress including PWV and ameliorates lipid profile and systemic and renal blood pressure without affecting glycemic levels, signifying the synergistic antioxidant potential and vasodilator action in pretreated diabetic SHRs.

Spontaneously hypertensive rats (SHRs) are more susceptible to diabetogenic effect of streptozotocin, most frequently used for the induction of diabetes [36] and causes increased production of reactive oxygen species (ROS) with activation of polyadenosine diphosphate ribosylation and nitric oxide release in SHRs [37].

Oxidative stress has been demonstrated in the pathogenesis of hypertension in SHR hypertension and diabetes. Vascular oxidative stress has been observed in different models of experimental hypertension like angiotensin II-induced hypertension, Dahl salt-sensitive hypertension, and obesity-associated hypertension and in SHR [38], lead-induced, salt-sensitive, and essential hypertension and diabetes mellitus [7]. Consequently, we attempted to develop a well-known rat model of a combined state of essential hypertension with diabetes.

Physiological and metabolic indices were kept into consideration to assess experimental diabetes in SHRs, including body weight, which was significantly reduced as one of the pronounced effects of STZ on *β*-cells [39]. Polyuria and polydipsia were also observed as significant metabolic perturbations of diabetic SHRs and found to be in accordance with the observations of Khan et al. [40]. As an acceptable explanation for the observed polydipsia among the rats with experimental early diabetic nephropathy as reported by others and us, this could be due to the fluid loss in the face of severe polyuria in these animals [41]. However, SHR+CNT only showed a decrease in fluid intake as compared to WKY+CNT. This could be due to the species variation, difference in sodium metabolism [42], and an overexpression of Ang-II, aldosterone [43], and vasopressin in this genetic model of hypertensive rats. Another explanation for this could be the decreased plasma concentration of adiponectin in SHRs as compared to normotensive (WKY) which causes an inhibitory effect on ADH secretion and retains fluid volume in the body, thus indirectly leading to a decrease in fluid intake in the nontreated/control group.

Hyperglycemia due to poor glycemic control is common in overt diabetes and is associated with dysfunctions of different organs, particularly the kidney, nerves, eye, heart, and blood vessels [44]. Hyperglycemia with elevated BP causes damage to the vascular endothelial cells with increased oxidative stress and vascular reactivity [45] and is considered a vital phenomenon of oxidative stress [46]. Diabetes associated with hyperglycemia modifies the endothelial function through a number of complex mechanisms including oxidative stress [47], glycation of protein and lipids [48], and activation of protein kinase C [49]. Similarly, the endothelial-dependent vasodilatation is impaired in different animal models of hypertension including spontaneous hypertensive rats and renovascular hypertension [50]. Therefore, ROS formation can be a direct consequence of hyperglycemia.

In our study, the glycemic level was not influenced after either irbesartan, adiponectin, or pioglitazone either single or combination treatment protocol. This probably corresponds with the type of the diabetic model using STZ similar to the human type 1 diabetic model. It is well known that endothelial dysfunction occurs in diabetic complications [6], associates with atherosclerotic progression [4], and elevates in oxidative stress. In diabetic SHRs, variable observation in terms of increased or decreased SBP and MAP is reported [51, 52], whereas in our study findings, the MAP and SBP values of diabetic SHRs were considerably higher, which could be due to the rapid destruction of nitric oxide (NO) in STZ-induced SHRs [53], although diminished NO bioactivity and bioavailability are key characteristics for arterial hypertension and endothelial disorders [54].

Moreover, we also observed that hyperglycemic SHRs exhibited decreased RCBP which supports previous observations of our laboratory findings in a diabetic model of rats [40, 51], which is probably due to stimulation of local Ang-II and intrarenal RAAS [55]. In our findings, three weeks of irbesartan (partial PPAR-*γ* agonist) in combination with adiponectin significantly reduced RCBP, SBP, and MAP values to a larger extent as compared to adiponectin and pioglitazone (full PPAR-*γ* agonist) either single or combination pretreatments, which could be possibly due to upregulation of PPAR-*γ* receptors besides an increase in production of nitric oxide (NO) [56, 57]. The significance of NO in the kidney vasculature cannot be ruled out which performs various pivotal roles including renal hemodynamic regulation, modulation of renal sympathetic neural activity, and inhibition of the tubular sodium reabsorptive mechanism [58]. We observed that irbesartan at 30 mg/kg/day caused a maximal dose for blockade of RAAS while its partial PPAR-*γ* agonistic activity also contributed to its BP reduction and renoprotective characteristics in nondiabetic SHRs as observed previously in our findings [59].

Nonetheless, regulation of MAP and vascular tone depends upon NO, which acts as an endothelium-derived molecule [60], whereas plasma adiponectin stimulates production of NO with reduction in sensitivity to Ang-II [61]. Adiponectin receptors (Adipo R1 and Adipo R2) in endothelial cells mediate adiponectin-induced phosphorylation of AMPK and eNOS which together lead to an increase in NO production [62]. In our findings, activation of PPAR-*γ* with partial and full agonists (irbesartan and pioglitazone), respectively, upregulates plasma adiponectin levels probably by stimulating the expression of proteins involved in adiponectin assembly, for instance, endoplasmic reticulum oxidoreductin-1 protein (Erol-L*α*) and adiponectin secretion such as disulfide-bond A oxidoreductase-like protein (DsbA-L) [63]; however, we did not measure these proteins in our experimental protocol.

Moreover, in our findings, the heart rate of STZ-induced SHRs remained higher which could be due to the SNS overactivation [64], whereas the hypertensive state correlates with SNS activity, which, therefore, is intricately involved with the initiation and progression of hypertension causing increases in the heart rate [65] and supports our values obtained in diabetic SHRs. Previous findings confirm the adiponectin existence in the cerebrospinal fluid [66], thus controlling and reducing the sympathetic nerve activity and heart rate [67], indicating that adiponectin is merely responsible for the reduction in the heart rate of diabetic SHRs treated groups.

### 4.1. Adiponectin Concentration in Plasma and Lipid Profile

Diabetes induced by high-dose STZ is similar to the human type 1 diabetic model [68]; thus, reduction of plasma adiponectin concentration with STZ administration would contribute to the diabetic condition of SHRs and is in agreement with findings of Thulé et al. [69]. Interestingly, in our experimental protocol, STZ-induced SHRs treated with pioglitazone for 3 weeks in combination with exogenous adiponectin significantly increased adiponectin levels as compared to the other sets of treatment. It is also evident that pioglitazone while acting as an agonist for PPAR-*γ* improves endothelial function [70], with BP reduction and lipid metabolism [71] via stimulating the production of plasma adiponectin [21] and reduction in vascular sensitivity in diabetic SHRs [20].

In addition, we also measured lipid contents of experimental diabetic and genetic models of hypertensive pretreated rats. Plasma triglyceride concentrations were higher in control SHRs as compared to control WKY during the treatment period, whereas STZ treatment aggravates the condition in a genetic model of hypertensive rats, leading to a further significant increase in plasma triglyceride, LDL, and total serum cholesterol, with a decrease in adiponectin and HDL plasma concentrations, indicating anthropometric and physiological disorders. Previous studies reveal that full-length adiponectin activates AMP-activated protein kinase (AMPK) phosphorylation [72] stimulating fatty-acid oxidation and glucose utilization by activating AMP-activated protein kinase, thus suppressing gluconeogenesis in the liver [14]. However, phosphorylation of AMPK regulates enzymes responsible for the synthesis of triglycerides and fatty acids with their transcription factors, thus constraining basal and oxidized low-density lipoproteins through NADPH oxidase inhibition in endothelial cells [73], eventually leading to a decrease in adipose tissue mass through activation of adiponectin receptors present mainly in lateral hypothalamic nuclei [74]. Therefore, PPAR-*γ* agonists used in our study probably influenced the gene expression responsible for lipid and carbohydrate metabolism without affecting glycemic levels in diabetic SHRs. In parallel to our study findings, a previous study proved that pioglitazone attenuated dyslipidemia in cyclosporine-induced hypertensive rats [27], whereas in another study, Hussein et al. proved that a much greater beneficial effect of a combination of rosiglitazone and telmisartan offered more improvement in serum TGs and adiponectin [75]. Interestingly, treating diabetic rats with exogenous adiponectin and pioglitazone as full PPAR-*γ* agonist produced significant attenuation of metabolic dysfunctions, as evidenced by the significant decrease in TC, TGs, and LDL, but an increase in HDL and adiponectin plasma concentrations as a similar conclusion was drawn for plasma adiponectin concentration.

### 4.2. Pulse Wave Velocity and Antioxidant Changes

Oxidative stress defines an imbalance between production of free radicals, its reactive metabolites, and so-called oxidants or reactive oxygen species (ROS), whereas their elimination is by protective mechanisms, referred to as antioxidants. This imbalance leads to damage of important biomolecules and cells, with potential impact on the whole organism [76]; however, oxidative stress and reactive oxygen derivatives further aggravate diabetes and hypertension [7]. In SHR, oxidative stress appears to be the cause of hypertension development on a larger scale, and the major effect of PPAR-*γ* activation is the reduction of oxidative stress levels [77]. Recent epidemiological studies together with human diabetic models have suggested an association between adiponectin concentration and oxidative stress; thus, decreased circulating adiponectin levels predominates in increased oxidative stress, which is closely linked with diabetic complications [1, 2, 3, 78] and a key feature of increased production of ROS and proinflammatory pathways [11]. Reactive oxygen species, including reactive nitrogen species, hydrogen peroxide, superoxide, and hydroxyl anions, are the most significant O_2_ derivate, which impacts vascular biology. Moreover, vascular fibrosis also determines the vascular structural modifications in extracellular matrix (ECM) components, collagen types I and III, elastin, and fibronectin [79]. Production of ROS reduces bioavailability of NO due to uncoupling of eNOS, with enhanced levels of superoxide anions leading to formation of peroxynitrite, thus aggravating the impairment of eNOS activity and reducing NO production [80]. In our experiment, an imbalance between antioxidants and oxidative stress was observed in diabetic SHRs, which can be confirmed from the increased plasma levels of free radical mediated products of lipid peroxidation (MDA), decreased plasma concentration of enzymatic antioxidant SOD, and nonenzymatic antioxidant GSH. A decrease in TAC further confirms this imbalance indicating free radical production with a weak antioxidant defence system in diabetic SHRs, signifying the importance of OS as a common denominator in all these pathways.

Arterial stiffness is linked with endothelial dysfunction, whereas the pulse wave velocity (PWV) is considered a surrogate marker (a well-established index for arterial stiffness) [25] and vascular diseases. The stiffer artery would lead to an increase in the PWV due to the persistent hyperglycemia leading to depletion of the antioxidant defence mechanism, generating free radicals [81] resulting in endothelial dysfunction and reduced vascular elasticity. Therefore, pulse wave velocity of diabetic SHRs was significantly higher as compared to control rats indicating the marked decrease in the extensibility of blood vessels in diabetic conditions leading to increased arterial stiffness.

However, we observed that exogenously administered adiponectin attenuated the arterial stiffness (PWV) of diabetic SHRs along with a decrease in SBP and MAP, which could be at least partially mediated through its potent antioxidant characteristics and was attenuated by blocking endothelial-derived nitric oxide synthase activity, suggesting that relaxant effect was possibly mediated by nitric oxide. However, the combination with pioglitazone resulted in a significantly greater decrease in PWV as compared with combined treatment of adiponectin with irbesartan and separate treatments. Previous clinical studies have demonstrated that partial PPAR-*γ* agonist (ARBs) protects vascular endothelium via an increase of endothelial NO synthesis [82] and plasma adiponectin concentration [57] thus preventing endothelial dysfunction more effectively as compared to non-PPAR-*γ* agonists ARBs [83]. Likewise, full PPAR-*γ* agonist, pioglitazone, stimulates the production of NO and moderates oxidative stress through activation of signaling cascades, such as cAMP-PKA and AMPK-eNOS component [70], and by increasing glutathione levels, thus supporting the fact that AMPK serves as a major downstream molecule for adiponectin production [65].

The data from this study will add to the understanding of the combined treatment of adiponectin with full but not partial PPAR-*γ* agonist in a combined model of hypertension and diabetes. The PPAR-*γ* ligands have other important effects that inhibit atherosclerosis, including (1) improvement of endothelial function, (2) attenuation of vascular cell growth and migration, (3) inhibition of major transcription pathways mediating vascular inflammation, and (4) increase of reverse cholesterol transport. The specific agonists of PPAR-*γ*, TZDs, have demonstrated protective effects on a variety of atherosclerosis biomarkers and on surrogate measures of CVD, in addition to improved conventional measures of CVD risk [84]. Full PPAR-*γ* agonist such as pioglitazone involves an adiponectin-dependent pathway, which increases adiponectin levels, ameliorating insulin resistance, increasing AMPK activation, and decreasing gluconeogenesis in the liver [19]. In contrary, angiotensin II receptor blockers may increase adiponectin production directly by activating the nuclear receptor PPAR-*γ* [24] as partial PPAR agonists in vitro and in vivo [23]. RAAS blockers increase plasma adiponectin levels better as compared to other antihypertensive agents [85]. It is, therefore, likely that partial agonists such as angiotensin II receptor blockers irbesartan act in a similar way. However, full PPAR-*γ* agonists (pioglitazone) not only act at the transcriptional level but also show to activate critical chaperones in the secretory pathway and to increase the release of the HMW form of adiponectin [86]. The use of blood pressure-lowering and antidiabetic agents in this study was capable of enhancing antioxidant potential through NO-dependent or NO-independent mechanisms in combination therapy of adiponectin with pioglitazone which was significantly higher as compared to separate and irbesartan combination therapy, which could be due to the difference in partial and full agonistic activity for PPAR-*γ* receptors, conferred by irbesartan and pioglitazone, respectively, and were therefore found to have greater beneficial/synergistic effects on the genetic model of hypertension with STZ-induced diabetes.

In this study, combined treatment of exogenous adiponectin with full PPAR-*γ* agonist (pioglitazone) significantly attenuates the oxidative status to a larger extent as compared to cotreatment of adiponectin with irbesartan in experimentally induced diabetic SHRs. There was a marked increase in NO, SOD, and TOC plasma levels that indicates improvement in arterial stiffness with decreased oxidative stress in diabetic SHRs. Similarly, the reduced lipid peroxidation (MDA) values denote a decrease in free radical production, thus substantiating our findings and supporting our hypothesis tested. Increased antioxidant levels (SOD and GSH) imply better defence against ROS. These antioxidants protect the cells from oxidative damage, thereby decreasing the oxidative stress-mediated vascular complications through antioxidant-mediated pathways.

## 5. Conclusion

In a nut shell, exogenous adiponectin administration attenuated the vascular abnormalities, fluctuating from endothelial dysfunction to ROS production, through nitric oxide and antioxidant enzymatic properties with abrogation of arterial stiffness. Nonetheless, owing to the full PPAR-*γ* agonist activity of pioglitazone, cotreatment with adiponectin significantly augmented to a larger extent with improvement in oxidative status and serum triglycerides and restoration of atrial stiffness (*in vivo* biomarker) with antioxidant enzymatic potential indicating a degree of synergism existing between adiponectin and pioglitazone.

## Figures and Tables

**Table 1:** Metabolic parameters of WKY, SHR control, and SHR diabetic treated groups with irbesartan, pioglitazone, adiponectin, and a combination of adiponectin with irbesartan or pioglitazone.

Parameters	Groups	Days of observation			
		Day 0	Day 8	Day 21	Day 28
Body weight (g)	WKY+CNT	245 ± 5	250 ± 7	275 ± 4^∗^	289 ± 8^∗^
	SHR+CNT	242 ± 3	248 ± 6^^^	267 ± 8^∗^	284 ± 9^∗^
	SHR+STZ	245 ± 3	200 ± 5^∗^^^^	208 ± 7^∗^^^^	209 ± 10^∗^
	SHR+STZ+Irb	250 ± 5	215 ± 3^∗^	211 ± 8^∗^	212 ± 6^∗^
	SHR+STZ+Pio	254 ± 6	205 ± 4^∗^	207 ± 7^∗^	217 ± 5^∗^^ж^
	SHR+STZ+Adp	252 ± 4	213 ± 7^∗^	215 ± 9^∗^	206 ± 4^∗^
	SHR+STZ+Irb+Adp	251 ± 7	208 ± 3^∗^	206 ± 8^∗^	204 ± 6^∗^
	SHR+STZ+Pio+Adp	247 ± 5	201 ± 9^∗^	200 ± 10^∗^	209 ± 5^∗^

Water intake (mL/d)	WKY+CNT	43 ± 1	44 ± 2	45 ± 3	44 ± 2
	SHR+CNT	32 ± 2^^^	34 ± 2^!^	34 ± 3^!^	37 ± 4^!^
	SHR+STZ	33 ± 2	48 ± 3^^^^∗^	48 ± 2^^^^∗^	59 ± 3^^^^∗^
	SHR+STZ+Irb	34 ± 2	48 ± 3^∗^	50 ± 2^∗^	50 ± 3^∗^
	SHR+STZ+Pio	35 ± 2	47 ± 2^∗^	51 ± 2^∗^	60 ± 3^∗^
	SHR+STZ+Adp	36 ± 2	46 ± 3^∗^	47 ± 2^∗^	57 ± 3^∗^
	SHR+STZ+Irb+Adp	35 ± 2	47 ± 2^∗^	49 ± 2^∗^	56 ± 3^∗^
	SHR+STZ+Pio+Adp	34 ± 2	45 ± 3^∗^	49 ± 2^∗^	55 ± 3^∗^

UFR (mL/min/100 g)	WKY+CNT	3.84 ± 0.44	3.63 ± 0.21	3.92 ± 0.21	3.95 ± 0.21
	SHR+CNT	3.10 ± 0.05^!^	2.98 ± 0.09^!^	2.94 ± 0.54^!^	2.93 ± 0.43^!^
	SHR+STZ	3.09 ± 0.04	12.35 ± 0.52^^^	12.79 ± 0.62^^^	13.18 ± 0.04^^^
	SHR+STZ+Irb	3.06 ± 0.02	12.57 ± 0.25^∗^	12.57 ± 0.37^^^^∗^	12.58 ± 0.22^∗^^#^
	SHR+STZ+Pio	3.07 ± 0.03	13.58 ± 0.55^∗^^*δ*^	13.57 ± 0.27^∗^^*δ*^	13.58 ± 0.30^∗^^*δ*ж^
	SHR+STZ+Adp	3.06 ± 0.02	13.28 ± 0.49^∗^^*δ*^	13.35 ± 0.15^∗^^*δ*^	16.25 ± 0.13^∗^^*δ*^
	SHR+STZ+Irb+Adp	3.08 ± 0.02	13.57 ± 0.39^∗^^*δ*^	13.35 ± 0.25^∗^^*δ*^	17.79 ± 0.15^∗^*^δΦ^*
	SHR+STZ+Pio+Adp	3.09 ± 0.03	13.57 ± 0.29^∗^^*δ*^	13.58 ± 0.24^∗^^*δ*^	20.28 ± 0.29^∗^*^δζ^*

Blood glucose (mg/dL)	WKY+CNT	89 ± 3	88 ± 2	86 ± 2	88 ± 3
	SHR+CNT	91 ± 3	90 ± 2	88 ± 3	89 ± 3
	SHR+STZ	90 ± 3	460 ± 18^∗^^^^	471 ± 14^∗^^^^	489 ± 25^∗^^^^
	SHR+STZ+Irb	89 ± 4	458 ± 11^∗^	462 ± 17^∗^	470 ± 16^∗^
	SHR+STZ+Pio	90 ± 5	471 ± 21^∗^	477 ± 19^∗^	474 ± 15^∗^
	SHR+STZ+Adp	88 ± 2	465 ± 19^∗^	462 ± 18^∗^	484 ± 27^∗^
	SHR+STZ+Irb+Adp	89 ± 2	488 ± 10^∗^	484 ± 16^∗^	479 ± 19^∗^
	SHR+STZ+Pio+Adp	86 ± 3	479 ± 21^∗^	486 ± 18^∗^	480 ± 22^∗^

Notes: the values are presented as the mean ± SEM (*n* = 6) in each group and were analyzed by repeated measures one-way ANOVA followed by *Bonferroni post hoc* test. Values with *P* < 0.05 were statistically significant during and at the end of treatment. ! indicates significant difference (*P* < 0.05) between the SHR and WKY control groups. ^ indicates significant difference (*P* < 0.05) between the WKY and SHR control groups in comparison to the SHR diabetic control group. ∗ indicates significant difference (*P* < 0.05) in comparison to day 0 of the respective group. *δ* indicates significant difference (*P* < 0.05) of the diabetic Irb, Pio, Adp, Irb+Adp, and Pio+Adp groups in comparison to the SHR diabetic control group. # indicates significant difference (*P* < 0.05) between the diabetic Irb and Adp groups. ж indicates significant difference (*P* < 0.05) between the diabetic Pio and Adp groups. *Φ* indicates significant difference (*P* < 0.05) of the diabetic Adp group in comparison to the diabetic Irb+Adp group at days 21 and 28. *ζ* indicates a significant difference (*P* < 0.05) of the diabetic Adp group in comparison to the diabetic Pio+Adp group at days 21 and 28.

**Table 2:** Systemic hemodynamic parameters of WKY, SHR control, and SHR diabetic treated groups with irbesartan, pioglitazone, adiponectin, and a combination of adiponectin with irbesartan or pioglitazone.

Parameters	Groups	Days of observation			
		Day 0	Day 8	Day 21	Day 28
Systolic blood pressure (mmHg)	WKY+CNT	118 ± 5	117 ± 2	117 ± 2	120 ± 3
	SHR+CNT	159 ± 4^!^	164 ± 6^!^	162 ± 3^!^	157 ± 8^!^
	SHR+STZ	161 ± 5^^^	173 ± 3^^^^∗^	177 ± 4^^^^∗^	175 ± 3^^^^∗^
	SHR+STZ+Irb	163 ± 4	177 ± 5^∗^	147 ± 4^∗^^*δ*^	135 ± 3^∗^^*δ*^
	SHR+STZ+Pio	165 ± 6	179 ± 4^∗^	155 ± 3^∗^^*δ*ж^	148 ± 6^∗^^*δ*ж^
	SHR+STZ+Adp	162 ± 4	175 ± 2^∗^	174 ± 3^∗^^#^	138 ± 4^∗^^*δ*^
	SHR+STZ+Irb+Adp	164 ± 3	176 ± 3^∗^	148 ± 4^∗^^*δ*^	118 ± 3*^δΦ^*
	SHR+STZ+Pio+Adp	163 ± 5	177 ± 4^∗^	154 ± 4^∗^^*δ*^	134 ± 3^∗^^*δ*^

Diastolic blood pressure (mmHg)	WKY+CNT	79 ± 3	84 ± 4	80 ± 3	86 ± 5
	SHR+CNT	119 ± 6^!^	117 ± 7^!^	108 ± 7^!^	120 ± 6^!^
	SHR+STZ	117 ± 3	119 ± 2	120 ± 3	118 ± 4
	SHR+STZ+Irb	118 ± 2	121 ± 3	101 ± 2^∗^^*δ*^	92 ± 1^∗^^*δ*^
	SHR+STZ+Pio	116 ± 3	119 ± 3	109 ± 5^∗^^*δ*^	106 ± 4^∗^
	SHR+STZ+Adp	117 ± 4	120 ± 4	118 ± 4^#ж^	96 ± 2^∗^^*δ*#ж^
	SHR+STZ+Irb + Adp	115 ± 4	118 ± 3	103 ± 3^∗^^*δ*^	88 ± 2^∗^*^δΦ^*
	SHR+STZ + Pio+Adp	118 ± 3	120 ± 2	111 ± 3^∗^^*δ*^	98 ± 2^∗^^*δ*^

Mean arterial pressure	WKY+CNT	92 ± 8	97 ± 4	92 ± 4	97 ± 5
	SHR+CNT	132 ± 5^!^	133 ± 4^!^	126 ± 5^!^	132 ± 6^!^
	SHR+STZ	132 ± 7	137 ± 5^^^^∗^	143 ± 4^^^^∗^	144 ± 5^^^^∗^
	SHR+STZ+Irb	133 ± 5	140 ± 6^∗^	118 ± 3^∗^^*δ*^	106 ± 4^∗^^*δ*^
	SHR+STZ+Pio	132 ± 4	139 ± 4^∗^	124 ± 4^∗^^*δ*^	120 ± 5^∗^^*δ*ж^
	SHR+STZ+Adp	132 ± 3	138 ± 7^∗^	137 ± 6^∗^	110 ± 8^∗^^*δ*^
	SHR+STZ+Irb+Adp	131 ± 4	137 ± 5^∗^	118 ± 4^∗^^*δ*^	98 ± 5*^δΦ^*
	SHR+STZ+Pio+Adp	133 ± 5	139 ± 5^∗^	125 ± 3^∗^^*δ*^	105 ± 6^∗^^*δ*^

Heart rate (BPM)	WKY+CNT	312 ± 10	310 ± 4	309 ± 11	303 ± 8
	SHR+CNT	386 ± 9^!^	390 ± 10^!^	392 ± 14^!^	389 ± 11^!^
	SHR+STZ	387 ± 5^!^^	394 ± 7^^^	402 ± 4^^^^∗^	407 ± 5^^^^∗^
	SHR+STZ+Irb	385 ± 4	396 ± 4^∗^	393 ± 4^∗^^*δ*#^	386 ± 3^*δ*#^
	SHR+STZ+Pio	388 ± 6	400 ± 3^∗^	380 ± 3^∗^^*δ*^	367 ± 4^∗^^*δ*ж^
	SHR+STZ+Adp	383 ± 3	398 ± 5^∗^	395 ± 4^∗^^ж^	356 ± 3^∗^^*δ*^
	SHR+STZ+Irb+Adp	386 ± 5	401 ± 4^∗^	396 ± 3^∗^^*δ*^	360 ± 4^∗^^*δ*^
	SHR+STZ+Pio+Adp	387 ± 7	403 ± 5^∗^	377 ± 6^∗^^*δ*^	351 ± 5^∗^^*δ*^

Notes: the values are presented as the mean ± SEM (*n* = 6) in each group and were analyzed by repeated measures one-way ANOVA followed by *Bonferroni* post hoc test. Values with *P* < 0.05 were considered statistically significant. ! indicates significant difference (*P* < 0.05) between the SHR and WKY control groups during and at the end of treatment. ∗ indicates significant difference (*P* < 0.05) in comparison to day 0 of the respective group. *δ* indicates a significant difference (*P* < 0.05) of the diabetic Irb, Pio, Adp, Irb+Adp, and Pio+Adp groups in comparison to the SHR diabetic control group. # indicates significant difference (*P* < 0.05) between the diabetic Irb and Adp groups during and at the end of treatment. ж indicates significant difference (*P* < 0.05) between the diabetic Pio and Adp groups during and at the end of treatment. *Φ* indicates significant difference (*P* < 0.05) of the diabetic Adp group in comparison to the diabetic Irb+Adp group at days 21 and 28.

**Table 3:** Plasma triglycerides and lipoprotein (LDL, HDL, and VLDL) level profile of WKY, SHR control, and SHR diabetic treated groups with irbesartan, pioglitazone, adiponectin, and a combination of adiponectin with irbesartan or pioglitazone.

Groups	Lipid profile			
	Triglycerides (mg/dL)	Total cholesterol (mg/dL)	HDL (mg/dL)	LDL (mg/dL)
WKY+CNT	50.75 ± 4.09	61.25 ± 2.29	16.33 ± 1.2	36.25 ± 1.8
SHR+CNT	84.80 ± 11.32^!^	149.96 ± 17.54^!^	67.64 ± 3.97^!^	97.08 ± 6.33^!^
SHR+STZ	172.4 ± 14.48^∗^	197.72 ± 12.72^∗^	42.02 ± 4.63^∗^	122.49 ± 6.01^∗^
SHR+STZ+Irb	153.9 ± 10.5^*δ*#^	166.84 ± 15.0^*δ*#^	59.78 ± 4.88^*δ*#^	108.56 ± 7.97^*δ*#^
SHR+STZ+Pio	147.2 ± 10.7*^δж^*	153.98 ± 11.64*^δж^*	72.89 ± 2.85*^δж^*	98.96 ± 7.50^*δ*^
SHR+STZ+Adp	93.20 ± 8.29^*δ*^	129.60 ± 12.19^*δ*^	79.22 ± 3.96^*δ*^	96.03 ± 5.20^*δ*^
SHR+STZ+Irb+Adp	85.25 ± 2.35^*δ*^	136.57 ± 5.7^*δ*^	79.25 ± 4.1^*δ*^	95.28 ± 4.5^*δ*^
SHR+STZ+Pio+Adp	73.25 ± 4.5*^δζ^*	119.25 ± 6.7*^δζ^*	77.28 ± 5.7^*δ*^	91.25 ± 5.4^*δ*^

Notes: the values are presented as the mean ± SEM (*n* = 6) in each group and were analyzed by one-way ANOVA followed by *Bonferroni post hoc* test. Values with *P* < 0.05 were considered statistically significant during and at the end of treatment. ! indicates significant difference (*P* < 0.05) between the SHR and WKY control groups. ∗ indicates significant difference (*P* < 0.05) in comparison to the SHR control group. *δ* indicates significant difference (*P* < 0.05) of the diabetic Irb, Pio, Adp, Irb+Adp, and Pio+Adp groups in comparison to the SHR diabetic control group. # indicates significant difference (*P* < 0.05) between the diabetic Irb and Adp groups. ж indicates a significant difference (*P* < 0.05) between the diabetic Pio and Adp groups. *ζ* indicates a significant difference (*P* < 0.05) of the diabetic Adp group in comparison to the diabetic Pio+Adp group at days 21 and 28.

## Data Availability

The analyzed data have been incorporated in the tables and figures of the manuscript, whereas the values for these analyses of the data have been provided in the supplementary files submitted with the manuscript.

## Supplementary Materials

Supplementary Materials Supplementary files contain the individual values on days of observation for each group and parameters observed in the study and were used statistically for data expression in the manuscript.
