# Supplementary material for: Antioxidant Potential of Adiponectin and Full PPAR-γ Agonist in Correcting Streptozotocin-Induced Vascular Abnormality in Spontaneously Hypertensive Rats
Source: PPAR Res. 2021 Oct 14;2021:6661181. doi: 10.1155/2021/6661181 (PMC8531825; doi:10.1155/2021/6661181)
Supplement: Supplementary Materials — Supplementary files contain the individual values on days of observation for each group and parameters observed in the study and were used statistically for data expression in the manuscript. [file 6661181.f1.pdf]

Parameters	Groups	Days of Observation			
		Day 0	Day 8	Day 21	Day 28
Body Weight (g)	WKY	1) 245<br>2) 250<br>3) 240<br>4) 245<br>5) 240<br>6) 250	1) 250<br>2) 255<br>3) 245<br>4) 240<br>5) 250<br>6) 260	1) 275<br>2) 279<br>3) 271<br>4) 270<br>5) 275<br>6) 280	1) 289<br>2) 300<br>3) 280<br>4) 280<br>5) 290<br>6)295
	SHR	1) 242<br>2) 245<br>3) 239<br>4) 238<br>5) 245<br>6) 240	1) 248<br>2) 245<br>3) 240<br>4) 255<br>5) 255<br>6) 245	1) 267<br>2) 275<br>3) 260<br>4) 258<br>5) 278<br>6) 264	1) 284<br>2) 293<br>3) 270<br>4) 280<br>5) 284<br>6) 295
	SHR+STZ	1) 245<br>2) 242<br>3) 248<br>4) 240<br>5) 246<br>6) 247	1. 200<br>2. 205<br>3. 198<br>4. 202<br>5. 204<br>6. 191	1) 208<br>2) 210<br>3) 200<br>4) 213<br>5) 217<br>6) 200	1) 209<br>2) 215<br>3) 197<br>4) 210<br>5) 223<br>6) 197
	SHR+STZ+Irb	1. 248<br>2. 244<br>3. 247<br>4. 256	1. 216<br>2. 220<br>3. 218<br>4. 213	1. 206<br>2. 200<br>3. 214<br>4. 221	1. 208<br>2. 203<br>3. 216<br>4. 221

		5. 255<br>6. 250	212<br>211	5. 217<br>6. 208	5. 217<br>6. 207
	SHR+STZ+Pio	1) 254<br>2) 260<br>3) 248<br>4) 248<br>5) 252<br>6) 262	1) 205<br>2) 209<br>3) 201<br>4) 201<br>5) 205<br>6) 211	1) 207<br>2) 214<br>3) 200<br>4) 200<br>5) 207<br>6) 217	1) 217<br>2) 222<br>3) 212<br>4) 212<br>5) 221<br>6) 223
	SHR+STZ+Adp	1) 252<br>2) 256<br>3) 248<br>4) 248<br>5) 252<br>6) 258	1) 213<br>2) 220<br>3) 206<br>4) 206<br>5) 213<br>6) 223	1) 215<br>2) 224<br>3) 206<br>4) 206<br>5) 212<br>6) 227	1) 206<br>2) 210<br>3) 202<br>4) 202<br>5) 206<br>6) 212
	SHR+STZ+Adp +Irb	250<br>263<br>256<br>249<br>247<br>241	1. 212<br>2. 211<br>3. 210<br>4. 208<br>5. 202<br>6. 205	1. 220<br>2. 212<br>3. 204<br>4. 202<br>5. 200<br>6. 198	1. 208<br>2. 196<br>3. 200<br>4. 198<br>5. 212<br>6. 210
	SHR+STZ+Adp +Pio	1) 247<br>2) 252<br>3) 242<br>4) 242<br>5) 247<br>6) 254	1) 201<br>2) 210<br>3) 192<br>4) 192<br>5) 201<br>6) 214	1) 200<br>2) 210<br>3) 190<br>4) 190<br>5) 200<br>6) 214	1) 209<br>2) 214<br>3) 204<br>4) 204<br>5) 209<br>6) 216

Water intake<br>(ml/d)	WKY	1) 43<br>2) 44<br>3) 42<br>4) 42<br>5) 42<br>6) 44	1) 44<br>2) 46<br>3) 42<br>4) 42<br>5) 44<br>6) 47	1) 45<br>2) 48<br>3) 42<br>4) 42<br>5) 44<br>6) 4	1) 45<br>2) 47<br>3) 43<br>4) 43<br>5) 45<br>6)48
	SHR	1) 32<br>2) 34<br>3) 30<br>4) 30<br>5) 32<br>6) 35	1) 34<br>2) 36<br>3) 32<br>4) 32<br>5) 34<br>6) 37	1) 34<br>2) 37<br>3) 31<br>4) 31<br>5) 34<br>6) 38	1) 37<br>2) 41<br>3) 33<br>4) 33<br>5) 37<br>6) 43
	SHR+STZ	1) 33<br>2) 35<br>3) 31<br>4) 31<br>5) 33<br>6) 36	1. 48<br>2. 51<br>3. 45<br>4. 45<br>5. 48<br>**6.** 52	1) 48<br>2) 50<br>3) 46<br>4) 46<br>5) 48<br>6) 51	1) 59<br>2) 62<br>3) 56<br>4) 56<br>5) 59<br>6) 63
	SHR+STZ+Irb	1) 38<br>2) 36<br>3) 35<br>4) 33<br>5) 30<br>6) 32	7) 53<br>8) 51<br>9) 49<br>10) 47<br>11) 45<br>12) 43	13) 54<br>14) 52<br>15) 50<br>16) 49<br>17) 48<br>18) 47	19) 46<br>20) 52<br>21) 49<br>22) 55<br>23) 48<br>24) 50
	SHR+STZ+Pio	1) 34<br>2) 36<br>3) 32<br>4) 32	1) 48<br>2) 51<br>3) 45<br>4) 45	1) 50<br>2) 52<br>3) 48<br>4) 48	1) 50<br>2) 53<br>3) 47<br>4) 47

		5) 34<br>6) 37	5) 48<br>6) 52	5) 50<br>6) 53	5) 50<br>6) 54
	SHR+STZ+Adp	1) 36<br>2) 38<br>3) 34<br>4) 34<br>5) 36<br>6) 39	1) 46<br>2) 49<br>3) 43<br>4) 43<br>5) 46<br>6) 50	1) 47<br>2) 49<br>3) 45<br>4) 45<br>5) 47<br>6) 50	1) 57<br>2) 60<br>3) 54<br>4) 54<br>5) 57<br>6) 61
	SHR+STZ+Adp +Irb	1) 39<br>2) 37<br>3) 35<br>4) 34<br>5) 33<br>6) 32	7) 44<br>8) 45<br>9) 46<br>10) 47<br>11) 49<br>12) 51	13) 46<br>14) 47<br>15) 48<br>16) 49<br>17) 53<br>18) 51	19) 51<br>20) 54<br>21) 55<br>22) 57<br>23) 59<br>24) 60
	SHR+STZ+Adp +Pio	1) 34<br>2) 36<br>3) 32<br>4) 32<br>5) 34<br>6) 37	1) 45<br>2) 48<br>3) 42<br>4) 42<br>5) 45<br>6) 49	1) 49<br>2) 51<br>3) 47<br>4) 47<br>5) 49<br>6) 52	1) 55<br>2) 58<br>3) 52<br>4) 52<br>5) 55<br>6) 59

UFR (mL/min/100 g)	WKY	1) 3.84<br>2) 4.28<br>3) 3.4<br>4) 3.4<br>5) 3.65<br>6) 4.45	1) 3.63<br>2) 3.84<br>3) 3.42<br>4) 3.42<br>5) 3.60<br>6) 3.93	1) 3.92<br>2) 4.13<br>3) 3.71<br>4) 3.71<br>5) 3.86<br>6) 4.22	1) 3.95<br>2) 4.16<br>3) 3.74<br>4) 3.74<br>5) 3.87<br>6) 4.25
	SHR	1) 3.10<br>2) 3.15<br>3) 3.05<br>4) 3.05<br>5) 3.10<br>6) 3.17	1) 2.98<br>2) 3.07<br>3) 2.89<br>4) 2.89<br>5) 2.98<br>6) 3.11	1) 2.94<br>2) 3.48<br>3) 2.4<br>4) 2.4<br>5) 2.73<br>6) 3.69	1) 2.93<br>2) 3.36<br>3) 2.5<br>4) 2.5<br>5) 2.80<br>6) 3.51
	SHR+STZ	1) 3.09<br>2) 3.13<br>3) 3.05<br>4) 3.05<br>5) 3.09<br>6) 3.15	1) 12.35<br>2) 12.84<br>3) 11.8<br>4) 11.8<br>5) 12.21<br>6) 13.06	1) 12.79<br>2) 13.41<br>3) 12.17<br>4) 12.17<br>5) 12.56<br>6) 13.64	1) 13.18<br>2) 13.22<br>3) 13.14<br>4) 13.14<br>5) 13.15<br>6) 13.23
	SHR+STZ+Irb	1) 3.6<br>2) 3.2<br>3) 3.1<br>4) 3.0<br>5) 2.6<br>6) 2.8	1. 13.6<br>2. 13.3<br>3. 12.9<br>4. 12.3<br>5. 11.8<br>6. 11.5	1) 11.8<br>2) 12.3<br>3) 11.5<br>4) 13.3<br>5) 12.9<br>6) 13.6	1) 13.3<br>2) 13.0<br>3) 13.6<br>4) 11.5<br>5) 12.3<br>6) 13.3
	SHR+STZ+Pio	1) 3.07<br>2) 3.1<br>3) 3.04<br>4) 3.04	1) 13.58<br>2) 14.08<br>3) 13.08<br>4) 13.08	1) 13.57<br>2) 13.77<br>3) 13.37<br>4) 13.37	1) 13.58<br>2) 13.88<br>3) 13.28<br>4) 13.28

		5) 3.07<br>6) 3.12	5) 13.43<br>6) 14.28	5) 13.52<br>6) 13.85	5) 13.49<br>6) 14.00
	SHR+STZ+Adp	1) 3.06<br>2) 3.08<br>3) 3.04<br>4) 3.04<br>5) 3.06<br>6) 3.09	1) 13.28<br>2) 13.77<br>3) 12.79<br>4) 12.79<br>5) 13.10<br>6) 13.95	1) 13.35<br>2) 13.50<br>3) 13.20<br>4) 13.20<br>5) 13.30<br>6) 13.55	1) 16.25<br>2) 16.38<br>3) 16.12<br>4) 16.12<br>5) 16.25<br>6) 16.43
	SHR+STZ+Adp +Irb	1) 3.0<br>2) 3.3<br>3) 3.6<br>4) 2.6<br>5) 2.8<br>6) 3.1	1) 13.3<br>2) 12.6<br>3) 12.4<br>4) 14.3<br>5) 14.9<br>6) 13.6	1) 14.7<br>2) 13.1<br>3) 14.1<br>4) 13.3<br>5) 12.1<br>6) 12.3	1) 19.0<br>2) 18.5<br>3) 18.2<br>4) 17.5<br>5) 17.1<br>6) 16.7
	SHR+STZ+Adp +Pio	1) 3.09<br>2) 3.12<br>3) 3.06<br>4) 3.06<br>5) 3.09<br>6) 3.14	1) 13.57<br>2) 13.86<br>3) 13.28<br>4) 13.28<br>5) 13.50<br>6) 13.98	1) 13.58<br>2) 13.82<br>3) 13.34<br>4) 13.34<br>5) 13.52<br>6) 13.92	1) 20.28<br>2) 20.57<br>3) 20.00<br>4) 20.00<br>5) 20.15<br>6) 20.68
Blood glucose (mg/dl)	WKY	1) 89<br>2) 92<br>3) 86<br>4) 90<br>5) 85<br>6) 92	1) 88<br>2) 90<br>3) 85<br>4) 89<br>5) 87<br>6) 90	1) 86<br>2) 87<br>3) 85<br>4) 89<br>5) 83<br>6) 86	1) 88<br>2) 90<br>3) 87<br>4) 84<br>5) 85<br>6) 92

	SHR	1) 91<br>2) 95<br>3) 89<br>4) 90<br>5) 94<br>6) 87	1) 90<br>2) 90<br>3) 87<br>4) 87<br>5) 91<br>6) 92	1) 88<br>2) 91<br>3) 84<br>4) 87<br>5) 86<br>6) 92	1) 89<br>2) 92<br>3) 89<br>4) 89<br>5) 89<br>6) 83
	SHR+STZ	1) 90<br>2) 93<br>3) 91<br>4) 89<br>5) 89<br>6) 84	1) 460<br>2) 478<br>3) 480<br>4) 450<br>5) 456<br>6) 432	1) 471<br>2) 460<br>3) 462<br>4) 465<br>5) 475<br>6) 498	1) 489<br>2) 514<br>3) 465<br>4) 465<br>5) 475<br>6) 523
	SHR+STZ+Irb	1) 83<br>2) 87<br>3) 88<br>4) 90<br>5) 92<br>6) 94	1) 472<br>2) 468<br>3) 462<br>4) 458<br>5) 446<br>6) 442	1) 483<br>2) 475<br>3) 467<br>4) 458<br>5) 449<br>6) 440	1) 446<br>2) 494<br>3) 478<br>4) 460<br>5) 468<br>6) 474
	SHR+STZ+Pio	1) 90<br>2) 95<br>3) 84<br>4) 84<br>5) 94<br>6) 94	1) 471<br>2) 492<br>3) 452<br>4) 444<br>5) 470<br>6) 497	1) 477<br>2) 496<br>3) 457<br>4) 454<br>5) 478<br>6) 500	1) 474<br>2) 489<br>3) 459<br>4) 455<br>5) 476<br>6) 492
	SHR+STZ+Adp	1) 88<br>2) 90<br>3) 86<br>4) 86	1) 465<br>2) 484<br>3) 446<br>4) 445	1) 462<br>2) 480<br>3) 444<br>4) 440	1) 484<br>2) 511<br>3) 457<br>4) 460

		5) 89<br>6) 91	5) 461<br>6) 491	5) 462<br>6) 484	5) 470<br>6) 522
	SHR+STZ+Adp +Irb	1) 93<br>2) 91<br>3) 90<br>4) 88<br>5) 87<br>6) 85	1) 500<br>2) 498<br>3) 490<br>4) 488<br>5) 478<br>6) 474	1) 504<br>2) 498<br>3) 490<br>4) 480<br>5) 472<br>6) 460	1) 508<br>2) 492<br>3) 484<br>4) 472<br>5) 464<br>6) 454
	SHR+STZ+Adp +Pio	1) 86<br>2) 89<br>3) 83<br>4) 82<br>5) 87<br>6) 89	1) 479<br>2) 500<br>3) 458<br>4) 454<br>5) 478<br>6) 505	1) 486<br>2) 504<br>3) 468<br>4) 462<br>5) 490<br>6) 506	1) 480<br>2) 502<br>3) 458<br>4) 454<br>5) 478<br>6) 508

Parameters	Groups	Days of Observation			Day 28
		Day 0	Day 8	Day 21	
**Systolic blood pressure (mmHg)**	WKY	1. 118	1. 117	1. 117	1. 116
		2. 127	2. 115	2. 119	2. 119
		3. 117	3. 116	3. 116	3. 123
		4. 112	4. 118	4. 118	4. 124
		5. 116	5. 121	5. 120	5. 120
		6. 119	6. 115	6. 114	6. 118
	SHR	1. 155	1. 165	1. 165	1. 150
		2. 166	2. 166	2. 166	2. 148
		3. 156	3. 171	3. 158	3. 166
		4. 161	4. 160	4. 161	4. 157
		5. 159	5. 169	5. 160	5. 156
		6. 158	6. 153	6. 164	6. 165
	SHR+STZ	1. 158	1. 173	1. 172	1. 177
		2. 165	2. 170	2. 175	2. 176
		3. 164	3. 176	3. 181	3. 174
		4. 169	4. 168	4. 174	4. 180
		5. 156	5. 175	5. 179	5. 173
		6. 157	6. 177	6. 182	6. 170
	SHR+STZ+Irb	1. 170	1. 185	1. 152	1. 141
		2. 165	2. 180	2. 150	2. 136
		3. 164	3. 178	3. 148	3. 135
		4. 163	4. 176	4. 147	4. 134
		5. 160	5. 175	5. 146	5. 133
		6. 156	6. 168	6. 139	6. 131
	SHR+STZ+Pio	1. 165	1. 175	1. 154	1. 138
		2. 166	2. 180	2. 155	2. 143
		3. 173	3. 185	3. 152	3. 153
		4. 156	4. 174	4. 153	4. 156
		5. 169	5. 179	5. 156	5. 151
		6. 161	6. 181	6. 160	6. 147
	SHR+STZ+Adp	1. 159	1. 177	1. 173	1. 135
		2. 166	2. 176	2. 170	2. 136
		3. 158	3. 174	3. 176	3. 144
		4. 161	4. 172	4. 171	4. 143
		5. 160	5. 173	5. 175	5. 139
		6. 168	6. 178	6. 179	6. 133
	SHR+STZ+Adp+Irb	1. 169	1. 181	1. 155	1. 123
		2. 166	2. 178	2. 150	2. 119
		3. 165	3. 177	3. 148	3. 118
		4. 164	4. 176	4. 147	4. 117
		5. 162	5. 174	5. 146	5. 116
		6. 158	6. 170	6. 142	6. 115
	SHR+STZ+Adp+Pio	1. 159	1. 172	1. 148	1. 132
		2. 166	2. 175	2. 155	2. 130
		3. 158	3. 181	3. 152	3. 137
		4. 165	4. 174	4. 153	4. 139
		5. 160	5. 179	5. 156	5. 135
		6. 171	6. 182	6. 160	6. 131

**Diastolic blood pressure(mmHg)**	WKY	1. 75<br>2. 77<br>3. 78<br>4. 79<br>5. 84<br>6. 82	1. 90<br>2. 87<br>3. 78<br>4. 80<br>5. 84<br>6. 85	1. 80<br>2. 77<br>3. 78<br>4. 79<br>5. 84<br>6. 82	1. 91<br>2. 92<br>3. 81<br>4. 80<br>5. 83<br>6. 90
	SHR	1. 110<br>2. 115<br>3. 119<br>4. 122<br>5. 124<br>6. 126	1. 107<br>2. 115<br>3. 113<br>4. 122<br>5. 125<br>6. 120	1. 106<br>2. 112<br>3. 98<br>4. 118<br>5. 105<br>6. 110	1. 112<br>2. 115<br>3. 120<br>4. 122<br>5. 124<br>6. 127
	SHR+STZ	1. 112<br>2. 117<br>3. 113<br>4. 121<br>5. 119<br>6. 120	1. 116<br>2. 117<br>3. 119<br>4. 122<br>5. 121<br>6. 120	1. 116<br>2. 117<br>3. 119<br>4. 122<br>5. 123<br>6. 124	1. 115<br>2. 117<br>3. 113<br>4. 121<br>5. 119<br>6. 124
	SHR+STZ+Irb	7. 121<br>8. 120<br>9. 119<br>10. 118<br>11. 116<br>12. 114	1. 126<br>2. 122<br>3. 123<br>4. 121<br>5. 119<br>6. 115	1. 104<br>2. 103<br>3. 102<br>4. 100<br>5. 99<br>6. 98	1. 94.5<br>2. 93.5<br>3. 92.5<br>4. 91.5<br>5. 90.5<br>6. 89.5
	SHR+STZ+Pio	1. 112<br>2. 115<br>3. 113<br>4. 121<br>5. 117<br>6. 118	1. 115<br>2. 117<br>3. 119<br>4. 122<br>5. 121<br>6. 120	1. 109<br>2. 112<br>3. 100<br>4. 115<br>5. 108<br>6. 110	1. 109<br>2. 103<br>3. 98<br>4. 108<br>5. 108<br>6. 110
	SHR+STZ+Adp	1. 111<br>2. 117<br>3. 113<br>4. 121<br>5. 119<br>6. 121	1. 114<br>2. 117<br>3. 119<br>4. 122<br>5. 123<br>6. 125	1. 115<br>2. 117<br>3. 113<br>4. 121<br>5. 119<br>6. 124	1. 94<br>2. 92<br>3. 95<br>4. 97<br>5. 98<br>6. 100
	SHR+STZ+Adp+Irb	1. 121<br>2. 118<br>3. 116<br>4. 115<br>5. 112<br>6. 108	1. 123<br>2. 121<br>3. 118<br>4. 117<br>5. 116<br>6. 113	1. 107<br>2. 105<br>3. 104<br>4. 103<br>5. 101<br>6. 98	1. 92<br>2. 90<br>3. 89<br>4. 87<br>5. 86<br>6. 84
	SHR+STZ+Adp+Pio	1. 115<br>2. 117<br>3. 113<br>4. 121<br>5. 119<br>6. 123	1. 118<br>2. 117<br>3. 119<br>4. 122<br>5. 121<br>6. 123	1. 106<br>2. 114<br>3. 111<br>4. 113<br>5. 115<br>6. 107	1. 97<br>2. 96<br>3. 99<br>4. 101<br>5. 95<br>6. 100
**Mean arterial pressure (mmHg)**	WKY	1. 84<br>2. 86	1. 92<br>2. 93	1. 87<br>2. 88	1. 90<br>2. 92

		3. 98<br>4. 102<br>5. 82<br>6. 100	3. 96<br>4. 97<br>5. 100<br>6. 104	3. 90<br>4. 92<br>5. 96<br>6. 99	3. 96<br>4. 97<br>5. 103<br>6. 104
	SHR	1. 129<br>2. 125<br>3. 130<br>4. 134<br>5. 135<br>6. 139	1. 130<br>2. 127<br>3. 132<br>4. 134<br>5. 135<br>6. 140	1. 120<br>2. 119<br>3. 124<br>4. 130<br>5. 131<br>6. 132	1. 127<br>2. 123<br>3. 130<br>4. 134<br>5. 135<br>6. 143
	SHR+STZ	1. 127<br>2. 122<br>3. 130<br>4. 134<br>5. 135<br>6. 144	1. 131<br>2. 134<br>3. 132<br>4. 137<br>5. 142<br>6. 146	1. 137<br>2. 139<br>3. 142<br>4. 145<br>5. 148<br>6. 147	1. 136<br>2. 141<br>3. 143<br>4. 146<br>5. 148<br>6. 150
	SHR+STZ+Irb	1. 140<br>2. 137<br>3. 135<br>4. 132<br>5. 129<br>6. 125	1. 148<br>2. 146<br>3. 142<br>4. 138<br>5. 135<br>6. 131	1. 123<br>2. 120<br>3. 119<br>4. 117<br>5. 116<br>6. 113	1. 113<br>2. 109<br>3. 107<br>4. 104<br>5. 103<br>6. 100
	SHR+STZ+Pio	1. 127<br>2. 128<br>3. 130<br>4. 134<br>5. 135<br>6. 138	1. 133<br>2. 135<br>3. 138<br>4. 139<br>5. 144<br>6. 145	1. 118<br>2. 122<br>3. 124<br>4. 125<br>5. 127<br>6. 128	1. 112<br>2. 116<br>3. 120<br>4. 122<br>5. 124<br>6. 126
	SHR+STZ+Adp	1. 128<br>2. 130<br>3. 132<br>4. 134<br>5. 132<br>6. 136	1. 132<br>2. 133<br>3. 135<br>4. 138<br>5. 142<br>6. 148	1. 131<br>2. 134<br>3. 132<br>4. 137<br>5. 142<br>6. 146	1. 98<br>2. 106<br>3. 110<br>4. 112<br>5. 114<br>6. 120
	SHR+STZ+Adp+Irb	1. 137<br>2. 133<br>3. 132<br>4. 131<br>5. 129<br>6. 124	1. 145<br>2. 140<br>3. 138<br>4. 137<br>5. 135<br>6. 127	1. 124<br>2. 120<br>3. 117<br>4. 119<br>5. 116<br>6. 112	1. 105<br>2. 104<br>3. 100<br>4. 96<br>5. 92<br>6. 91
	SHR+STZ+Adp+Pio	1. 130<br>2. 127<br>3. 132<br>4. 134<br>5. 135<br>6. 140	1. 133<br>2. 135<br>3. 138<br>4. 139<br>5. 144<br>6. 145	1. 120<br>2. 122<br>3. 124<br>4. 126<br>5. 128<br>6. 130	1. 98<br>2. 100<br>3. 102<br>4. 106<br>5. 108<br>6. 116
	WKY	1. 295	1. 302	1. 292	1. 290

**HR (beat/min)**		2. 306<br>3. 312<br>4. 316<br>5. 318<br>6. 325	2. 306<br>3. 308<br>4. 310<br>5. 314<br>6. 316	2. 304<br>3. 309<br>4. 310<br>5. 313<br>6. 326	2. 299<br>3. 303<br>4. 304<br>5. 308<br>6. 314
	SHR	1. 376<br>2. 380<br>3. 382<br>4. 384<br>5. 394<br>6. 400	1. 376<br>2. 380<br>3. 390<br>4. 394<br>5. 398<br>6. 402	1. 374<br>2. 386<br>3. 388<br>4. 390<br>5. 398<br>6. 416	1. 376<br>2. 380<br>3. 384<br>4. 392<br>5. 396<br>6. 406
	SHR+STZ	1. 380<br>2. 382<br>3. 384<br>4. 386<br>5. 388<br>6. 402	1. 376<br>2. 388<br>3. 390<br>4. 392<br>5. 394<br>6. 424	1. 396<br>2. 398<br>3. 402<br>4. 403<br>5. 405<br>6. 408	1. 400<br>2. 405<br>3. 406<br>4. 407<br>5. 409<br>6. 415
	SHR+STZ+Irb	1. 392<br>2. 388<br>3. 385<br>4. 383<br>5. 382<br>6. 380	1. 402<br>2. 398<br>3. 397<br>4. 396<br>5. 395<br>6. 388	1. 398<br>2. 395<br>3. 394<br>4. 393<br>5. 392<br>6. 386	1. 392<br>2. 387<br>3. 386<br>4. 385<br>5. 384<br>6. 382
	SHR+STZ+Pio	1. 379<br>2. 386<br>3. 387<br>4. 388<br>5. 392<br>6. 396	1. 396<br>2. 398<br>3. 399<br>4. 400<br>5. 401<br>6. 406	1. 376<br>2. 378<br>3. 379<br>4. 380<br>5. 381<br>6. 386	1. 360<br>2. 365<br>3. 367<br>4. 368<br>5. 369<br>6. 373
	SHR+STZ+Adp	1. 378<br>2. 381<br>3. 382<br>4. 383<br>5. 385<br>6. 389	1. 390<br>2. 396<br>3. 398<br>4. 399<br>5. 400<br>6. 405	1. 389<br>2. 393<br>3. 394<br>4. 395<br>5. 396<br>6. 403	1. 351<br>2. 354<br>3. 356<br>4. 357<br>5. 358<br>6. 360
	SHR+STZ+Adp+Irb	1. 395<br>2. 387<br>3. 386<br>4. 385<br>5. 384<br>6. 379	1. 408<br>2. 400<br>3. 402<br>4. 401<br>5. 399<br>6. 396	1. 390<br>2. 395<br>3. 396<br>4. 397<br>5. 398<br>6. 400	1. 365<br>2. 362<br>3. 361<br>4. 360<br>5. 358<br>6. 354
	SHR+STZ+Adp+Pio	1. 376<br>2. 386<br>3. 387<br>4. 388<br>5. 389<br>6. 396	1. 396<br>2. 401<br>3. 402<br>4. 403<br>5. 404<br>6. 412	1. 367<br>2. 375<br>3. 376<br>4. 377<br>5. 381<br>6. 386	1. 344<br>2. 349<br>3. 350<br>4. 351<br>5. 355<br>6. 357

**Parameters**	**Groups**	Triglycerides (mg/dL)	Total cholesterol (mg/dL)	HDL (mg/dL)	LDL (mg/dL)
Triglycerides and lipoproteins	WKY+CNT	56.2	64	18.6	38.5
		54.3	63	18	38
		52.5	62	16.5	36
		50.3	61	16	35.5
		46.2	59.5	14.5	35
		45	58	14.4	34.5
	SHR+CNT	98	173	73	106
		92	165	68.5	100
		88	154	68	99
		86	145	66.5	98
		80	135	65.5	94
		65	127	62	88
	SHR+STZ	190	214	50	130
		184	208	41	124
		178	200	43	122
		168	194	42	120
		160	188	40	126
		154	180	36	112
	SHR+STZ+Irb	168	187	66	120
		162	180	62	114
		156	170	64	108

		154	162	60	106
		145	155	58	104
		138	146	54	98
	SHR+STZ+Pio	160	170	75	108
		156	162	74.5	104
		150	158	72.5	103
		144	152	73.5	99
		138	144	71.5	92
		134	138	68	88
	SHR+STZ+Adp	104	146	84	103
		100	138	79	98
		96	132	78	95
		92	126	81	96
		86	120	82	97
		82	112	74	87
	SHR+STZ+Irb+Adp	88	145	84	102
		86	140	79	98
		85	138	78	96
		84	136	81	94
		87	132	82	93
		82	129	74	89
	SHR+STZ+Pio+Adp	79	130	86	97
		76	121	79	95

		74	120	77	86
		73	118	78	93
		72	116	76	92
		65	111	70	84

**Parameters**	**Groups**	Triglycerides (mg/dL)	Total cholesterol (mg/dL)	HDL (mg/dL)	LDL (mg/dL)
Triglycerides and lipoproteins	WKY+CNT	56.2	64	18.6	38.5
		54.3	63	18	38
		52.5	62	16.5	36
		50.3	61	16	35.5
		46.2	59.5	14.5	35
		45	58	14.4	34.5
	SHR+CNT	98	173	73	106
		92	165	68.5	100
		88	154	68	99
		86	145	66.5	98
		80	135	65.5	94
		65	127	62	88
	SHR+STZ	190	214	50	130
		184	208	41	124
		178	200	43	122
		168	194	42	120
		160	188	40	126
		154	180	36	112

	SHR+STZ+Irb	168	187	66	120
		162	180	62	114
		156	170	64	108
		154	162	60	106
		145	155	58	104
		138	146	54	98
	SHR+STZ+Pio	160	170	75	108
		156	162	74.5	104
		150	158	72.5	103
		144	152	73.5	99
		138	144	71.5	92
		134	138	68	88
	SHR+STZ+Adp	104	146	84	103
		100	138	79	98
		96	132	78	95
		92	126	81	96
		86	120	82	97
		82	112	74	87
	SHR+STZ+Irb+Adp	88	145	84	102
		86	140	79	98
		85	138	78	96
		84	136	81	94

		87	132	82	93
		82	129	74	89
	SHR+STZ+Pio+Adp	79	130	86	97
		76	121	79	95
		74	120	77	86
		73	118	78	93
		72	116	76	92
		65	111	70	84
